# Network analysis of exploratory behaviors of mice in a spatial learning and memory task

**DOI:** 10.1371/journal.pone.0180789

**Published:** 2017-07-10

**Authors:** Yusuke Suzuki, Itaru Imayoshi

**Affiliations:** 1 Medical Innovation Center/SK Project, Graduate School of Medicine, Kyoto University, Kyoto, Japan; 2 Graduate School of Biostudies, Kyoto University, Kyoto, Japan; 3 The Hakubi Center, Kyoto University, Kyoto, Japan; 4 Institute for Frontier Life and Medical Sciences, Kyoto University, Kyoto, Japan; 5 World Premier International Research Initiative–Institute for Integrated Cell-Material Sciences, Kyoto University, Kyoto, Japan; 6 Japan Science and Technology Agency, Precursory Research for Embryonic Science and Technology, Saitama, Japan; Tokai University, JAPAN

## Abstract

The Barnes maze is one of the main behavioral tasks used to study spatial learning and memory. The Barnes maze is a task conducted on “dry land” in which animals try to escape from a brightly lit exposed circular open arena to a small dark escape box located under one of several holes at the periphery of the arena. In comparison with another classical spatial learning and memory task, the Morris water maze, the negative reinforcements that motivate animals in the Barnes maze are less severe and less stressful. Furthermore, the Barnes maze is more compatible with recently developed cutting-edge techniques in neural circuit research, such as the miniature brain endoscope or optogenetics. For this study, we developed a lift-type task start system and equipped the Barnes maze with it. The subject mouse is raised up by the lift and released into the maze automatically so that it can start navigating the maze smoothly from exactly the same start position across repeated trials. We believe that a Barnes maze test with a lift-type task start system may be useful for behavioral experiments when combined with head-mounted or wire-connected devices for online imaging and intervention in neural circuits. Furthermore, we introduced a network analysis method for the analysis of the Barnes maze data. Each animal’s exploratory behavior in the maze was visualized as a network of nodes and their links, and spatial learning in the maze is described by systematic changes in network structures of search behavior. Network analysis was capable of visualizing and quantitatively analyzing subtle but significant differences in an animal’s exploratory behavior in the maze.

## Introduction

Many kinds of maze tasks are widely used to study the neural circuit structure and function underlying spatial reference memory [1–5]. In addition, there is an increasing demand for the development of high-throughput assay systems that can precisely detect cognitive defects in animal models of neural diseases, such as Alzheimer's disease (AD) [6,7]. The Morris water maze test has been widely used to evaluate spatial learning and memory in rodents [8–10]. As swimming skill affects performance in the water maze, and swimming sometimes induces stress in the subject mice [11,12], the Barnes maze is an appropriate alternative.

The Barnes maze was originally developed by Carol A. Barnes in 1979 for use with rats [1] and was later adapted for mice [3–5]. During the task, mice are placed in the middle of a circular area surrounded by multiple holes at the periphery and receive negative reinforcement. Bright lights in an exposed environment are typically used as negative reinforcement, and air jets or loud buzzing noises are sometimes used to increase the motivation of mice to undertake the task [13,14]. Mice try to escape to a dark box hidden underneath one of the peripheral holes. After repeated trials, mice can learn the location of the target hole by using spatial reference cues mounted outside the arena and consequently navigate the maze more efficiently. It has been shown that the Barnes maze can be used to evaluate spatial reference memory and learning, since the removal of cues from the maze environment causes a dramatic decrease in task performance [6,15,16]. Compared with the Morris water maze test, despair and anxiety behaviors are infrequently observed in the Barnes maze test, indicating the modest nature of the negative reinforcement [1,17–19].

The Barnes maze protocol is composed of three phases: habituation, training, and the probe test. In the habituation phase, the mice are introduced to the environment and task. In the training phase, mice are given numerous trials to learn the task, typically for a period of 5 days to a few weeks. Acquisition of spatial memory in the training phase is evaluated by the decrease in the number of errors (visiting dummy holes), latency time, and travel distance to reach the target hole. The probe test, which is typically done 24 h after the last training trial, is conducted to confirm that the mice retain the spatial reference memory that was acquired during the training phase. In the probe test, the mice explore the arena without the escape box. It is assumed that mice that remember the location of the target hole will have a shorter latency to reach the original target location, remain there longer, and search non-target hole locations less intensely.

Recent progress in neural circuit imaging, such as the development of miniature brain endoscopes [20–22], allows us to observe the neural circuit activity in freely moving animals. Further, neuronal populations and circuits of interest can be manipulated by utilizing optogenetic techniques [23–25]. These technical advances will contribute to the identification of neural circuit structures and functions involved in spatial reference memory. Although it is easier to combine the Barnes maze with these methods than the water maze, some Barnes maze procedures may potentially interfere with these applications. For example, when mice are released into the maze, a subject mouse is placed inside an opaque or black cylinder and then manually placed in the center of the maze. Although the camera cables or optical fibers are securely connected to the mouse’s head, such apparatuses can potentially interfere with smooth release. Furthermore, the release location can fluctuate as a result of inconsistent placement by experimenters. Use of the start box is one way to more stably release mice.

To provide another option for the smooth and stable release of mice, we have developed a start-lift and equipped a maze with it. In this LIFT entry method, the subject mouse is automatically raised up by the lift and released into the maze, so that it can start navigating the maze smoothly from exactly the same position across trials.

In traditional Barnes maze data analysis, the total number of errors, latency time, and travel distance to reach the target hole are measured, and these scores are used as indices of spatial learning and memory [1,3]. Some studies have tried to detect the search strategies of mice and found progressive changes in these strategies as the acquisition of spatial memory becomes established [1,3,26]. However, these conventionally used analyses are not always sufficient to reveal spatio-temporal dynamics in behavioral changes in memory formation, maintenance, and retrieval processes. To detect the neural circuit structure and plasticity underlying spatial learning and memory, it is critical to visualize the nature of exploratory behaviors and their dynamic changes during the maze task trials. In this study, to visualize the detailed behavioral patterns of mice exploring the maze, we developed a network analysis method for the analysis of Barnes maze data. The network analysis allowed us to visualize the exploratory behaviors of both individual mice and groups of mice as interconnected network structures, and various quantitative features consisting of the search network were provided. Furthermore, spatial learning could be described as systematic temporal changes in the network structures.

## Materials & methods

### Subjects

The subjects were naïve, 5- to 11-week-old male C57BL/6J mice (Japan SLC, Shizuoka, Japan). The mice were group housed in a standard laboratory environment, maintained on a 14-h light/10-h dark cycle at a constant temperature (23–24°C) and relative humidity (40–50%). Food (pellets; Japan SLC) and water were provided *ad libitum*. The present behavioral testing was done during the light phase. The experiments were approved by the Animal Care Committee of Kyoto University (permit numbers: Med Kyo 14126, Med Kyo 15503, and Med Kyo 16216) and conformed to all relevant regulatory standards. After the behavioral experiments, all mice were sacrificed by cervical dislocation.

### Measurement and control system

**Fig 1:**
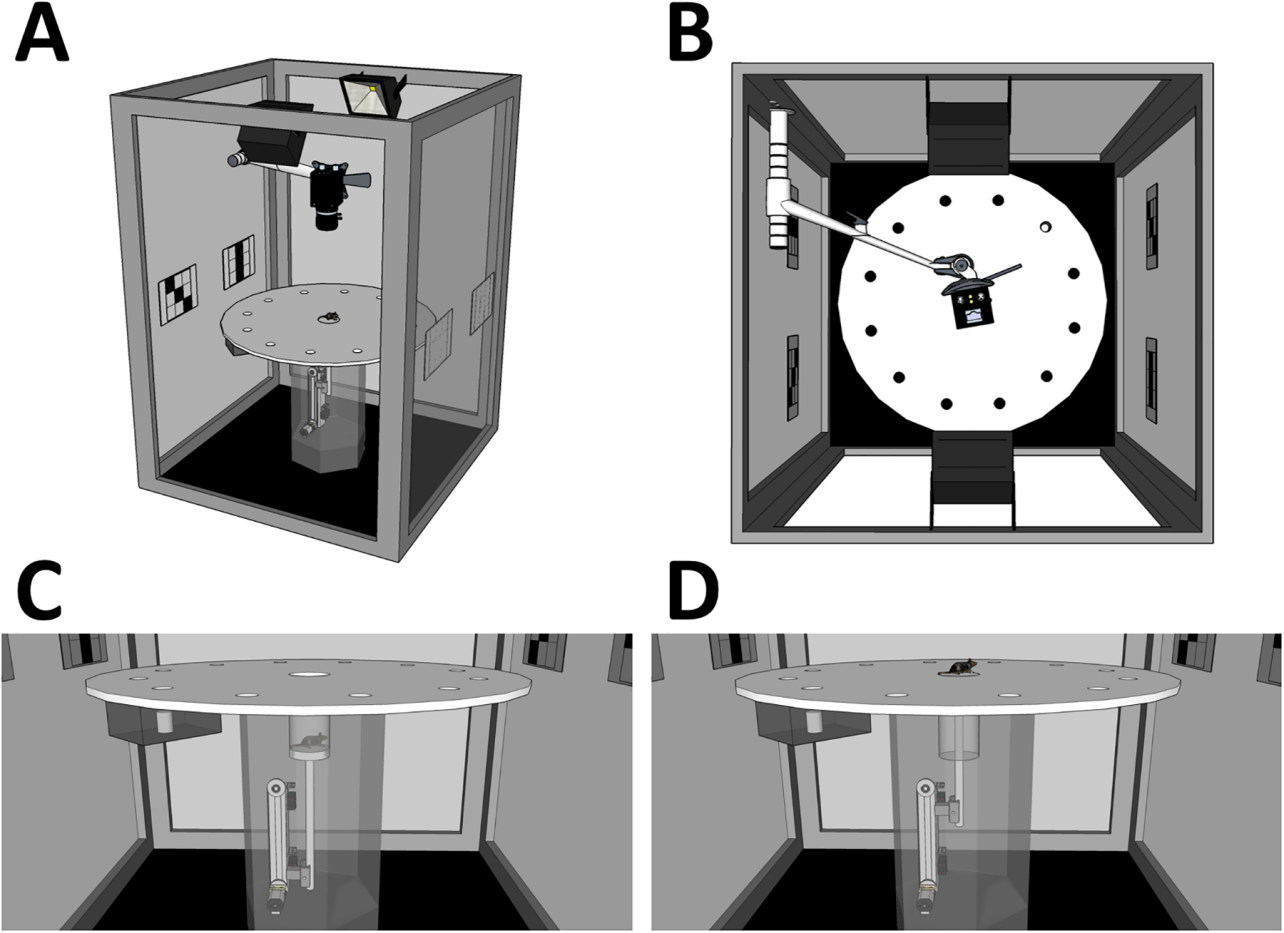
Design of the Barnes circular maze with a start lift. (A–D) Four views of the Barnes circular maze apparatus (overview, top view, side view before task start in the LIFT entry [see main text], and side view at the task start). The scales are proportional to the actual apparatuses except for the recording camera and the camera arm. Both items were enlarged for visibility.

The behavioral task (Barnes circular maze test, see below) was conducted on “dry land”: a white circular surface, 98 cm in diameter composing a custom-made Barnes maze apparatus (Bio-Medica, Osaka, Japan). The circular open arena was elevated 72 cm from the floor (Fig 1A–1D). Twelve holes were equally spaced around the perimeter at a distance of 40 cm from the centroid: the diameter of each hole was 4 cm. A black iron escape box (17 × 14 × 7 cm), which had paper cage bedding on its bottom, was located under one of the holes. This hole represented the target, analogous to the hidden platform in the Morris water maze task, and the remaining holes were considered dummies of the target. The location of the target was consistent for a given mouse, but was randomized across mice.

The entire apparatus was set within a cube-shaped outer enclosure (130 × 130 × 180 cm) with black curtains in order to obscure the outside scene of the arena and limit background noise. Two white color projection LED lights were mounted on the ceiling of the enclosure to ensure uniform and intense illumination of the arena (600 lx on the arena surface). A4-size (21.0 × 29.7 cm) unique spatial cues were mounted on each corner of the enclosure at a height of 86 cm from the floor. This apparatus was equipped with a start-lift at the center of the arena surface (Fig 1C and 1D). The scaffold of the lift (10 cm diameter) was made of the same material as the arena and was held 20 cm below the surface of the arena before the initiation of each trial. At the beginning of each trial, the lift transported a mouse to the arena surface (LIFT entry). The vertical movement of the lift was programmed so that the experimenter could control it externally at any time. In the MANUAL entries, the arena was changed to a circular platform without the start-lift. The remaining features of the platform were identical to those used in the LIFT entry task.

Behavior during the trials was recorded using a GigE Vision camera (UI-5240SE-NIR; IDS Imaging Development Systems GmbH, Obersulm, Germany) and displayed on a monitor outside of the outer enclosure. The camera was mounted on the ceiling of the enclosure (93.5 cm above the maze center), which could achieve a spatial resolution of 2 mm/pixel. Each image frame (500 × 500 pixels, with ×2 binning) was acquired at the rising edge on a 5-Hz pulse counter and sent to a main computer. All programs used for data acquisition, processing, and saving, as well as synchronized device controls, were written in LabVIEW 2013 (National Instruments, TX, US).

### Barnes circular maze test procedure

The protocol was the same as that used in previous studies [19,27–29]. Briefly, the protocol comprised three phases: habituation on Day 0, training from Days 1 to 6, and the probe test on Day 7. Mice were moved from the housing room to the experimental room more than 30 min before the experiment. They stayed in their home cages on a standby-rack in the experimental room while they were waiting for each trial. Immediately before the trial, a mouse was picked up from the cage with an opaque acrylic cylinder and released into the lift or directly onto the center of the arena. In the case of LIFT entries, mice were released into the lift while it was in the down position and were then lifted up to the arena surface simultaneously with the start of recording. In the case of MANUAL entries, mice were directly released around the center of the arena from the cylinder. Before the experiment, each mouse was assigned to either LIFT (n = 19) or MANUAL (n = 21) entry in a pseudo-random manner, and this assignment was constant across all experimental phases.

In the habituation phase, mice were allowed to freely explore the arena for 5 min. After that, they were moved to the escape box via the cylinder. After they had spent 5 min in the escape box, they were returned to the home cage. This procedure was executed once per mouse. In the training phase, mice explored the arena until they entered the escape box. Once the experimenter confirmed that the mouse had successfully entered the escape box, the recording was turned off. Each mouse was subjected to three training trials per day. Twenty-four hours after the last day of training (Day 6), mice were subjected to the probe test. In the probe test, mice explored the arena without the escape box for 3 min. This trial was done once per mouse. The maze was thoroughly cleaned with 70% ethanol solution and dried after every trial.

### Data analysis

The mouse position coordinates (x, y) for every recorded frame were estimated by three tracking algorithms. First, a shape adaptive mean shift algorithm (NI VISION 2013; National Instruments) was applied to detect the putative body of a mouse in the frame. When it failed to detect the mouse, an ellipse detection algorithm was then applied. If both failed (< 7% for all trials), a Tracking-Learning-Detection (TLD) algorithm [30] was applied *post hoc*. The image and point sequences throughout the trial were saved as movie and text files, respectively.

The shape adaptive mean shift and ellipse detection algorithms were applied online during the task, and the TLD algorithm was applied offline after the task if necessary. Due to the differences in the method of mouse release between the MANUAL and LIFT entries, all of the MANUAL trials but only 7% of the LIFT entry trials required offline tracking processing. During preprocessing for the offline tracking, several initial frames of image sequences of the MANUAL entry trials had to be discarded because undesired images of experimenters were captured just after mouse release. Then, a template of the mouse’s shape was manually selected in the first frame, and the mouse position was estimated in each of the subsequent frames. All programs for data analysis were written in MATLAB R2014b (MathWorks Inc., MA, USA). For each point sequence a coordinate transformation was applied so that the target holes for all mice were located at the top right. Conventional, strategy, and network analyses (see below) were then performed to extract the corresponding behavioral features. The definitions of these features are given below.

### Conventional analysis

**Fig 2:**
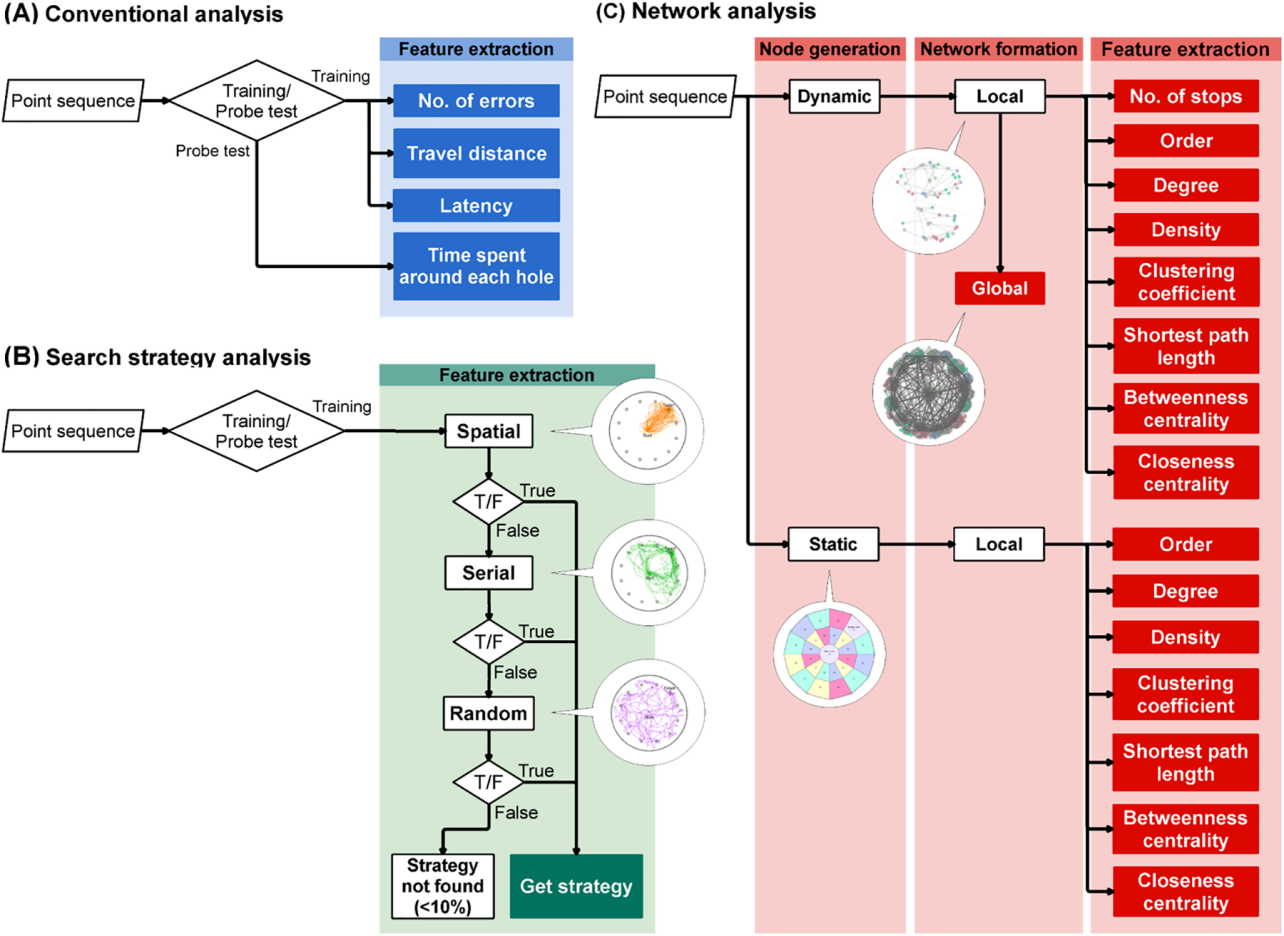
Processing flows of the conventional, search strategy, and network analyses. The processing flows of the conventional (A), strategy (B), and network (C) analyses are illustrated. The analysis started from the input of point sequences, represented by the parallelogram symbol, and then executed along the arrows. The diamond and rectangle symbols represent conditional branch and particular processing, respectively. Similar processing steps are included in the larger rectangles with the processing labels on the top. The filled symbols represent outcomes. The balloons indicate representative images of processing definitions or outputs. For instance, the images in the balloons in the strategy analysis (B) indicate representative trajectories of each strategy depicted in Fig 3A. Similarly, the bottom image in the network analysis (C) indicates the static zones and nodes shown in Fig B in S2 File. The top image indicates an example of the local network, and the middle image is a sample of the global network shown (see the Materials & Methods).

For the conventional analysis, the number of errors, time of latency, distance covered to reach the target hole, and time spent around each hole were extracted as behavioral features [19,27–29] (Fig 2A). The average of each feature across the three trials per day was calculated for each mouse.

### Search strategy analysis

The strategy analysis was carried out to find dynamic components of spatial learning (Fig 2B). Point sequences in each trial were categorized as a spatial, serial, or random strategy by algorithm-based classification. The definition of each strategy was based on previous studies [1,3,26] (Fig 2B, Table 1). In brief, if a mouse in a trial moved directly toward the target hole and the number of visits to dummy holes was less than three, the strategy was classified as “spatial”. The “serial” strategy was assigned when the mouse sequentially approached neighboring holes until they reached the target with fewer than three quadrant crossings. All remaining behavioral patterns were assigned the random strategy. The strategy analysis was applied to the data only in the training phase.

**Table 1:** Summary and abbreviations for behavioral features.

Analysis	Feature	Definition
**Conventional**	No. of errors	Sum of the number of visits to any area within 40 pixels (≈ mouse’s body length) from the centroid of the dummy holes.
	Latency (s)	Latency to enter the escape box.
	Travel distance (pixels)	Sum of the norms of the moving vector between two successive frames of a single trial movie.
	Time spent around each hole (frames)	Sum of the number of frames in which a mouse occupied an area within 40 pixels from the centroid of each hole.
**Strategy**	Spatial	If the movement history during a trial satisfied the following conditions: Number of quadrant crosses = 0, Number of errors < 3.
	Serial	If the movement history during a trial satisfied the following conditions: Number of quadrant crosses < 3, Order of hole visits was sequential, The strategy was not classified as Spatial.
	Random	If the movement history during a trial satisfied the following condition: The strategy was not classified as either Spatial or Serial.
**Network**	No. of stops (*o*)	The number of stopping coordinates in the network.
	Order (*n*)	The number of nodes bundling stopping coordinates in the network.
	Degree (*m*)	The number of links connected to a node in the network.
	Density (*ρ*)	The ratio of the number of links that were actually present in a network to the number of links that were theoretically possible in the network.
	Clustering coefficient (*C*_WS_)	The probability that two neighbors of a given node are themselves neighbors.
	Shortest path length (*l*)	The path such that no shorter path exists in the network, and its length is quantified as the number of links traversed along the shortest path between any two nodes.
	Betweenness centrality (*x*)	The extent to which a given node lies on the shortest path between other nodes.
	Closeness centrality (*cl*)	The inverse of the sum of a path length from a given node to other nodes.

The behavioral parameters and features analyzed in the conventional, strategy, and network analyses.

In the MANUAL entry trials, the initial positions of each mouse were distributed over a wide area (15.25 cm diameter on average; Fig A in S2 File, dark blue dashed circle). This is probably a result of the preprocessing of these data, because several initial frames of the image sequences were discarded (as described above) and the “initial” mouse positions differed from the original release positions. In contrast, in the LIFT entry trials, the initial mouse positions covered a more restricted area (4.52 cm diameter on average; Fig A in S2 File, dark red dashed circle) around the center of the maze. Therefore, more intact data were preserved in the LIFT entry method.

To avoid overestimation such that the random strategy was assigned even to micromovements within the lift scaffold, in the analysis of the LIFT entry, all points before the mouse initially exceeded a threshold were discarded. The threshold was set as the mean distance from the center in the MANUAL entry. The daily strategy usage for each mouse was determined by summing the frequency of each strategy used over the three trials performed that day.

### Network analysis

Network analysis was performed to determine if a particular structure co-occurred with each search strategy (Fig 2C). We expected that this structure would be expressed as a network represented by a set of nodes and links. In this analysis, the nodes were generated in two independent ways: dynamic and static. A history of transitions between nodes was also determined per trial, and each transition was treated as an undirected link between any two nodes. We did not presume any self-links in the network analysis.

In the dynamic node generation, a node was generated at the coordinates of each point where a particular action occurred [31]. In the present analysis, stopping behavior was picked as a representative action, and other criteria were also based on a previous analysis [31]. When the traveling distance within five successive frames was less than the threshold, one stopping coordinate was generated at the centroid of the points during the frames. The threshold was set at 20 pixels, which was approximately half of the average mouse’s body length. Then, a node was generated on a centroid of stopping coordinates set using the City Clustering Algorithm (CCA) [31,32]. In short, the CCA recursively processed data using the following three steps until a convergence condition was satisfied. First, the CCA selected the coordinates of a stopping point and then integrated those coordinates into the nearest node when a distance of less than 20 pixels between the stop and node was reached. If a stop was not integrated into any node under this criterion, the stop was treated as a new node. Second, the coordinates of all nodes were calculated, and each node was represented by the centroid of its constituent stops. Third, the CCA determined whether all stopping coordinates were integrated into a node. If not, the processing returned to the first step. Eventually, each node had unique coordinates, and the results were independent of the seed values (S1 Movie).

The original set of criteria in the dynamic node generation was developed for the analysis of the exploratory behavior in the rat [31]. However, in the present analysis, it was not certain that appropriate networks would be formed using the same criteria, since mice are generally more active than rats. Hence, in addition to the dynamic node network analysis, we conducted a network analysis using static nodes. Here, a total of 25 static nodes were uniformly scattered across the arena (Fig B in S2 File, “+” markers). The surrounding area of each node was designated as the specific zone of that node (Fig B in S2 File, colored zones). In the network analysis using these static nodes, a sequence of points during a trial was converted to a sequence of the nearest static nodes. Note that each network in the static node network analysis was a planar network where all nodes were connected only to nodes on adjacent zones [33].

The dynamic and static node data were analyzed separately. The network structure of each mouse was characterized by a total of eight features: (1) number of stops, (2) number of nodes (order), (3) degree, (4) density, (5) clustering coefficient, (6) shortest path length, (7) betweenness centrality, and (8) closeness centrality (Table 1). The definition of each feature conformed to previous studies [33,34]. Briefly, the number of stops (*o*) and order (*n*) were calculated by simply counting the stopping coordinates and nodes in the network, respectively. The degree (*m*) measured the number of links connected to a node in the network. The total degree was obtained using $m=\frac{1}{2}\sum_{ij}A_{ij}$, where A is an adjacency matrix whose element *A*_*ij*_ records the presence of a link between nodes *i* and *j*. If *A*_*ij*_ = 1, there is a link from node *i* to *j* and is zero otherwise. The average degree in the network was calculated by dividing it by the order *n* in the network. The density (*ρ*) is the ratio of the number of links that are actually present *m* in a network to the number of links that are theoretically possible in the network. The number of possible links was calculated by $\frac{1}{2}n(n-1)$. Thus, density is expressed as $\rho =\frac{2m}{n(n-1)}$, where *n* is the order of the network. The clustering coefficient (*C*_*ws*_) is the probability that two neighbors of a given node are themselves neighbors. This was calculated by dividing the total number of pairs of neighbors of node *i* by the number of pairs of neighbors linking to each other. The denominator is determined by $\frac{1}{2}k_{i}(k_{i}-1)$, where *k*_*i*_ is the degree of node *i*. The numerator is $\frac{1}{2}k_{i}R_{i}$, where *R*_*i*_ is *redundancy*, which is the average number of links from a neighbor of node *i* to other neighbors of *i*. Thus, the clustering coefficient of node *i* is expressed as $C_{i}=\frac{R_{i}}{k_{i}-1}$. The average of the clustering coefficient in the entire network is given by $C_{WS}=\frac{1}{n}\sum_{i=1}^{n}C_{i}$, where *n* is the order of the network. Between two nodes in the network, the shortest path (*l*) was quantified as the number of links traversed along the shortest path between the two. Each shortest path could be solved using Dijkstra's algorithm. The average shortest path length across nodes in the network was calculated. Betweenness centrality (*x*) is the extent to which a given node lies on the shortest paths between other nodes and is given by $x_{i}=\sum_{st}\frac{n_{st}^{i}}{g_{st}}$. If node *i* lies on the shortest path from node *s* to node *t*, $n_{st}^{i}$ is 1, otherwise it is 0. The sum of these values is then divided by *g*_*st*_ in which is the total number of shortest paths from *s* to *t*. The average was calculated across all nodes in the network. Closeness centrality (*cl*) measures the inverse of the sum of path lengths from a given node to other nodes. The closeness centrality of node *i* is expressed as ${cl}_{i}=\frac{1}{\sum_{j}d_{ij}}$, where *d*_*ij*_ is the path length from node *i* to *j*. The average closeness centrality across all nodes in the network was calculated.

One mouse in one group formed one network per trial; thus, all mice comprising one group represented the group network each day. The former network was local, whereas the latter was a global network (Figs 2C, 6A and 6B). In the present analysis, we also visualized the structures of global networks with the same method that we used to define the dynamic nodes. Nodes in a global network were determined by using the CCA for the coordinates of all nodes in all local networks in a single day. The integration threshold was the same as the dynamic threshold used to form local networks (see above). Once all local nodes belonged to any single global node, the global links were identified between the global nodes.

### Scopolamine treatment

Thirty-seven naïve mice were subjected to the Barnes circular maze test using the protocol described above. Scopolamine hydrobromide (Sigma-Aldrich Co. LLC, MO, USA) was prepared as a 0.3 mg/ml stock solution in 0.9% saline. Then, 3 mg of scopolamine hydrobromide/kg of body weight was injected intraperitoneally 20 min before starting the probe test. Nineteen mice were assigned to the scopolamine group (SCOP), whereas 18 mice were assigned to the vehicle-only group (VEH). This experiment was conducted with the LIFT entry method, and the data were not integrated for a comparison between the MANUAL and LIFT entries.

### Statistical analysis

For the analysis of the data of the MANUAL and LIFT entries, in the conventional analysis, a two-way (Entry × Day) analysis of variance (ANOVA) was applied for each parameter. If significant differences were detected in either or both of the interaction and/or main effects, Tukey’s honest significant difference (HSD) test was performed for multiple comparisons. Pearson’s *R* was calculated to indicate effect size. The magnitude of the effect size was conventionally classified as null (0 ≤ *R* < 0.1), small (0.1 ≤ *R* < 0.3), medium (0.3 ≤ *R* < 0.5), or large (0.5 ≤ *R* ≤ 1).

In the strategy analysis, the Kruskal–Wallis test was applied to look for differences in the usage of each strategy between the entry types (MANUAL and LIFT) in each day. The Friedman test was applied for the usage of each strategy across days within entries and for the usage of strategies within entries within days. Since significant differences were detected in the Friedman test, multiple comparison tests were applied. Paired comparison using the sign test was performed under the adjusted significance level given by Ryan’s procedure.

In the network analysis, the Kruskal–Wallis test and the Friedman test were applied for features between entries per day and for features across days within entries, respectively. Multiple comparison tests, with an adjusted significance level per the Ryan procedure, were applied when significant differences were detected. Paired comparison using the Mann–Whitney test was performed in the Kruskal–Wallis test, whereas the sign test was used in the Friedman test. For all statistical analyses, the significance level was set at 0.05.

To analyze the data of the scopolamine treatment, the same statistical methods described above were applied to look for differences between the VEH and SCOP groups.

## Results

### Conventional analysis for MANUAL and LIFT entries

**Fig 3:**
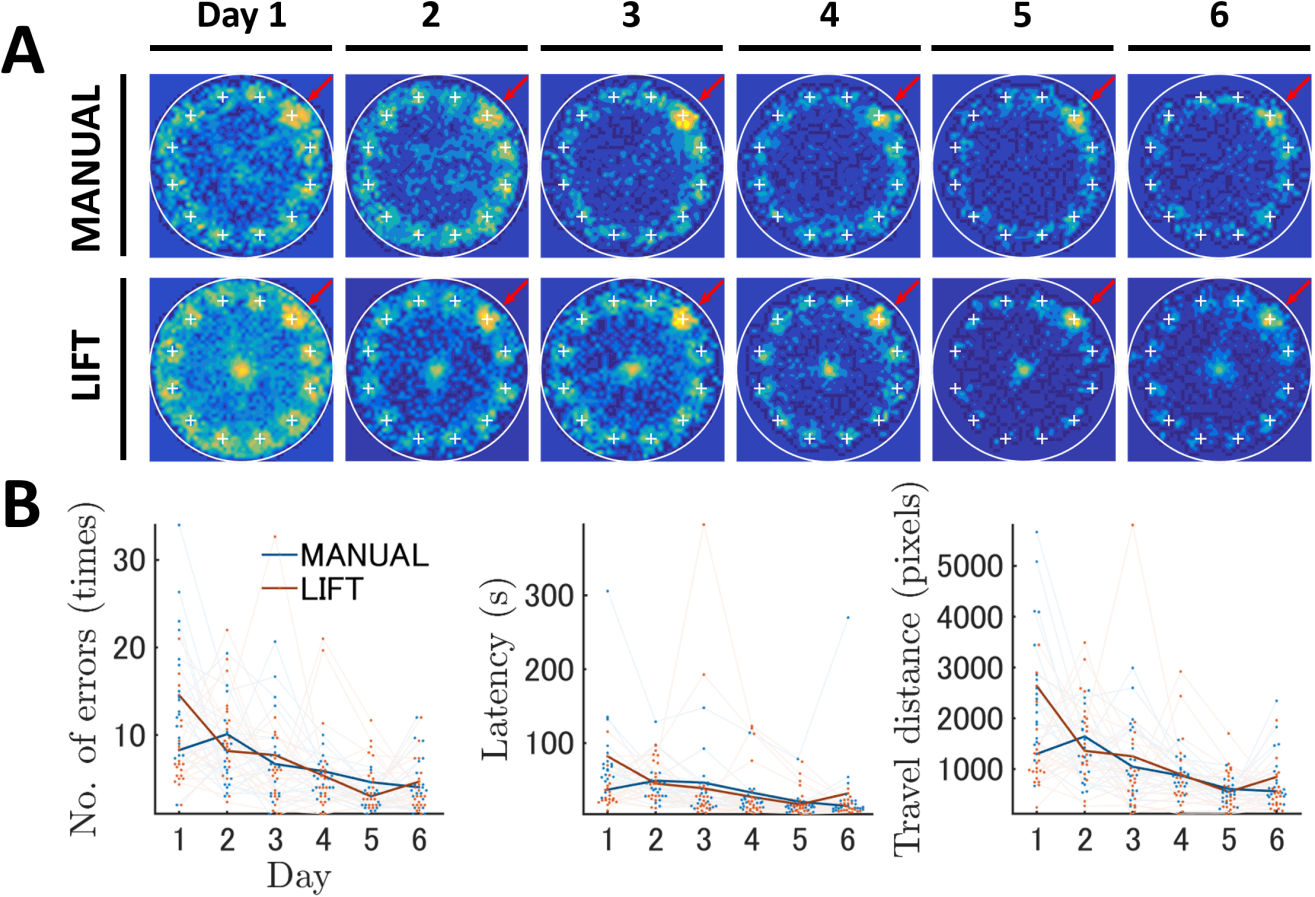
Analysis of the Barnes maze data from the MANUAL and LIFT entries using the conventional analysis method. Conventional analysis revealed that spatial learning was generated in both of the LIFT and the MANUAL entries during the training phase. (A) Changes in the occupancy rate across training. The occupancy rates in each block of the field are expressed by the contour map. The top and bottom rows indicate the results of the MANUAL and LIFT entry groups, respectively. Each column indicates the corresponding training day. The occupancy rate is graded by a color map ranging from cold to warm colors. Large white circles indicate the field edge. The plus markers indicate the locations of holes. The hole markers highlighted by the red arrows are the target locations. (B) Changes in conventional features across training. The number of errors, latency, and travel distance to reach the target are shown. The dark blue and dark red lines represent the mean results of the MANUAL and LIFT entries, respectively. The value of and change in the individual mouse data are illustrated by dots and light lines.

First, we analyzed and compared the data for MANUAL and LIFT entries with the conventional method that is widely used in Barnes maze experiments. In the analysis of the training phase, the total number of errors, the latency time, and the travel distance to reach the target hole were measured for each trial of an individual mouse. These scores were averaged in blocks of three trials per day and analyzed by two-way mixed-design ANOVA (Fig 3B, Table A in S1 File).

In both the MANUAL and LIFT entry data, it was apparent that all scores gradually decreased over the training period (Fig 3B), indicating spatial learning was successfully generated. Although the general trends of the learning curves of the MANUAL and LIFT groups were similar as judged by these three parameters, we found significant differences between the two groups. Our analysis with two-way ANOVA for each parameter detected a significant interaction between entry and day (Table A in S1 File): number of errors (*F*_5,190_ = 4.70, *p* < 0.05), latency (*F*_5,190_ = 4.21, *p* < 0.05), and travel distance (*F*_5,190_ = 6.47, *p* < 0.05). Tukey's HSD *post hoc* test reported that all three scores on Day 1 for number of errors, latency, and travel distance were significantly higher in the LIFT than in the MANUAL entry condition (Table B in S1 File). It was likely that, on Training Day 1, mice released by the start-lift explored and spent a longer time near the start position than the manually released mice (Fig 3A). However, after Training Day 2, both groups of mice vigorously explored the peripheral regions and rapidly left the center of the arena (Fig 3A). Indeed, we did not detect any significant difference in the number of errors, latency, or travel distance from Days 2 to 6 in either group.

As mentioned previously, accompanying spatial learning during the training phase, mice of both the MANUAL and LIFT entry groups showed significant reductions in the scores of the number of errors, latency, and travel distance. In the MANUAL entry group, significant decreases were observed in the number of errors between Days 1 and 6 and Days 2 and 4–6 (*p* < 0.05 each, Table C in S1 File). The latency to reach the goal was reduced between Days 2 and 5 and 6 and Days 3 and 6 (*p* < 0.05, Table C in S1 File). The travel distance was significantly shortened between Days 1 and 5 and 6 and Days 2 and 4–6 (*p* < 0.05 each, Table C in S1 File). In the LIFT entry group, the number of errors exhibited significant decreases between Days 1 and 2–6, Days 2 and 5, and Days 3 and 5 (*p* < 0.05 each, Table C in S1 File). The latency was significantly shortened among Days 1 and 2–6 (*p* < 0.05 each, Table C in S1 File). Similarly, a significant reduction in travel distance was observed between Days 1 and 2–6, Days 2 and 5, and Days 3 and 5 (*p* < 0.05 each, Table C in S1 File).

**Fig 4:**
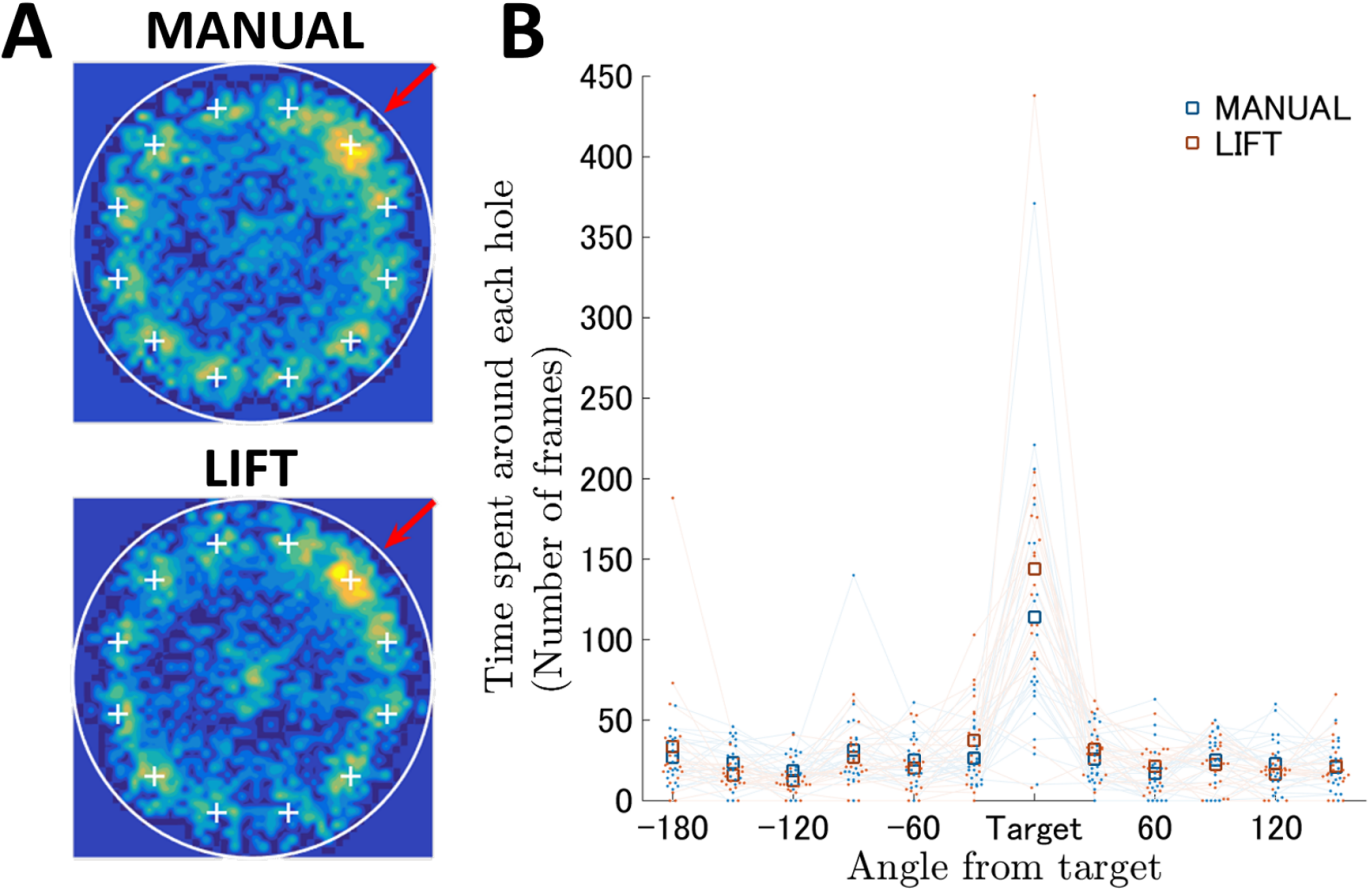
The probe test for spatial reference memory in the Barnes maze with MANUAL and LIFT entries. (A) The occupancy rates in each block of the field are shown on the contour map. The results of the MANUAL and LIFT entry groups are shown. The definitions of the color code and field markers are the same as those in Fig 3A. (B) Prolonged searching time near the target hole in the LIFT entry. The horizontal axis indicates the locations of the holes expressed as angle differences from the target. The vertical axis indicates the search time for the individual hole. The blue and red squares represent the mean results of the MANUAL and LIFT entries, respectively. The value of and change in each individual mouse’s data are shown as dots and light lines. Mice in both entry groups spent significantly more time near the target hole compared with the other holes. The LIFT group mice spent slightly, but significantly, more time near the target hole compared with the MANUAL group mice (see also Table E in S1 File), indicating that more precise spatial reference memory was generated in the LIFT entry than in the MANUAL entry.

Twenty-four hours after the last day of training, a probe test for reference memory was conducted to confirm that proficiency in this spatial task had been acquired based on navigation using distal environment cues, rather than other solutions, such as sequential checking. Mice in both entry groups approached the target hole and spent significantly more time near the target hole compared with the other holes (Fig 4A and 4B; Table D in S1 File). The two-way ANOVA for duration of time spent near each hole showed a significant interaction between entry and hole (*F*_11,418_ = 1.18, *p* < 0.05, Table D in S1 File). Both entry groups spent significantly more time around the target than around other holes (*p* < 0.05, Table F in S1 File). The LIFT group mice spent slightly, but significantly, more time near the target hole compared with the MANUAL group mice (Table E in S1 File). Therefore, the behavioral scores for spatial learning and reference memory determined using the conventional data analysis were similar to those found using our semi-automated Barnes circular maze equipped with the start-lift.

### Strategy analysis

**Fig 5:**
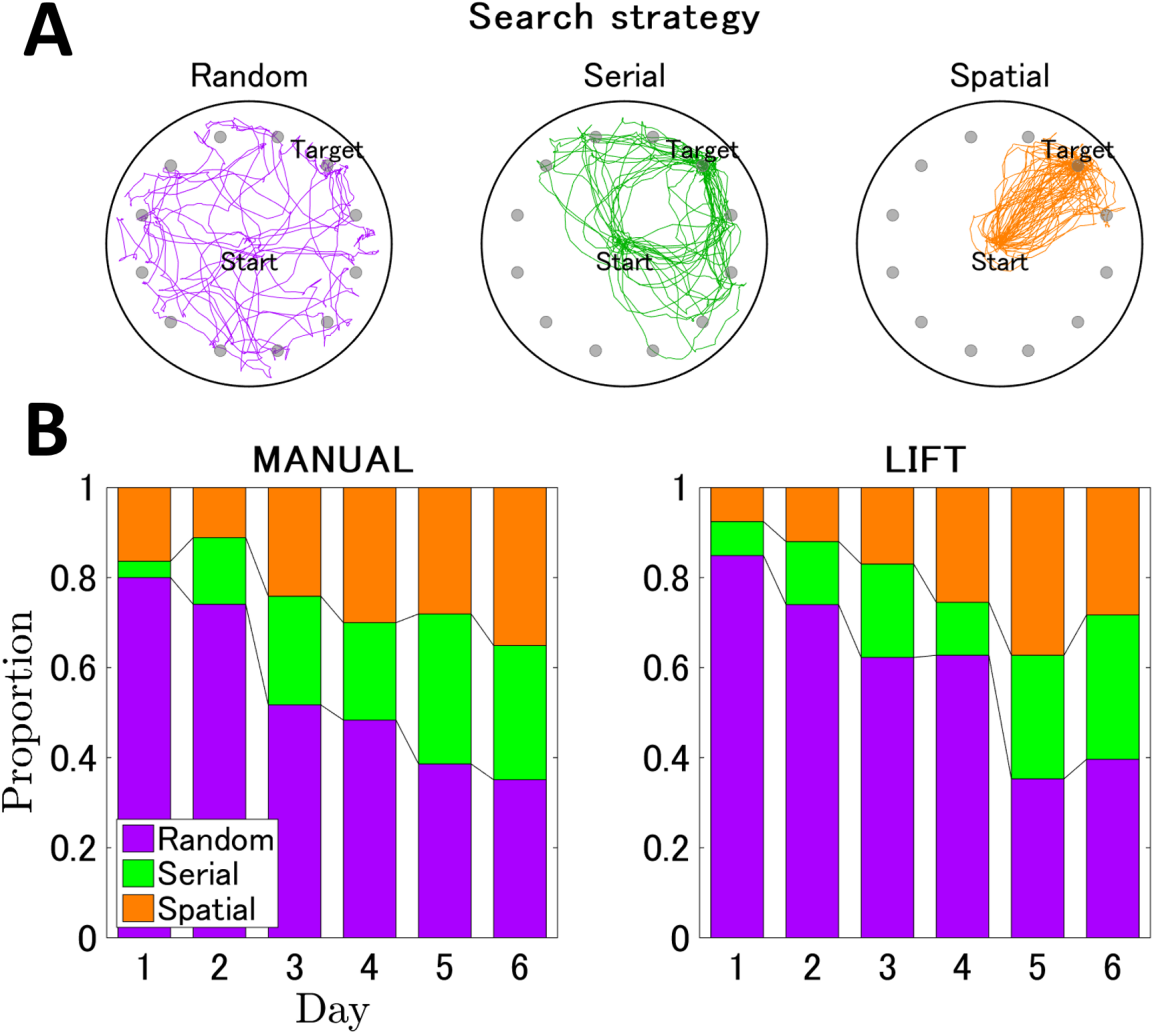
Changes in search strategies accompanied by spatial learning. (A) Examples of representative trajectories of the random, serial, and spatial strategies. The random, serial, and spatial strategies are shown in violet, green, and orange trajectories, respectively. The trajectory of one mouse per day of trials is shown; all trajectories were transformed so that the goal is located at the right top hole, noted as “Target”. The number of trajectories displayed in each panel was adjusted so that the total distance of trajectories is comparable between the three strategies. (B) The normalized stacked bar graphs indicating the ratios of strategy usage each day for the MANUAL entry (left panel) and the LIFT entry (right panel). The vertical and horizontal axes indicate the proportion of the strategy usage and the training days, respectively. Each bar represents the ratio of the strategy in a particular training day and is normalized so that the sum of each strategy ratio is 1. Temporal changes in usage within each strategy across training trials seemed to be more clearly observable for the LIFT than for the MANUAL entry group (see also Tables H–K in S1 File).

To assess the qualitative aspects of spatial learning, we analyzed the respective search strategies used by the mice to locate the target hole (Figs 2B and 5A) [1,3,26]. The search strategies were originally described by Barnes [1] and the detailed definition of each strategy used in this study was described in the Materials & Methods. The random search strategy includes unorganized searching of the maze with many center crossings (Figs 2B and 5A, violet). The serial strategy is characterized by the mouse running to the perimeter and visiting consecutive holes in a clockwise or counterclockwise manner (Figs 2B and 5A, green). The spatial strategy is the most efficient search strategy and involves moving directly to the target hole or to an adjacent hole before visiting the target (Figs 2B and 5A, orange).

Mice in both the MANUAL and LIFT entry groups initially employed the random strategy and showed a similar decrease in the use of this strategy across trials. The use of the random strategy decreased to <40% by the last training day. In both entry types, mice shifted to the serial and spatial strategies. This shift decreased the number of errors, latency time, and travel distance covered to reach the target hole (see Fig 5B). Although the Kruskal–Wallis test for a difference in the probability of using each strategy between MANUAL and LIFT entries showed no significant difference for all days (Table G in S1 File), the search strategy use shifted differently between the two groups. The Friedman test reported significant differences between strategy usages until Day 3 for MANUAL entry (*p* < 0.05, Table H in S1 File) and until Day 4 for LIFT entry (*p* < 0.05, Table H in S1 File). In the LIFT entry group, usage of the random strategy was highest from Days 1 to 3 (*p* < 0.05, Table I in S1 File); the usage became evenly split between strategies after Day 5. In contrast, in the MANUAL entry group, the dominance of the random strategy lasted until Day 2 (*p* < 0.05, Table I in S1 File) and the usages of all strategies were comparable after Day 4.

Temporal changes in usage within each strategy across training trials seemed to be more clearly observable for the LIFT than for the MANUAL entry group (Fig 5B). The Friedman test revealed that a significant increase in the usage of the spatial strategy across days was detected only in the LIFT entry group (χ^2^ (5) = 18.41, *p* < 0.05, Table J in S1 File), whereas significant changes in the random and serial strategies were commonly observed in both groups (*p* < 0.05, Table J in S1 File). We further statistically analyzed and multiply compared the temporal changes in each strategy (Table K in S1 File). In the MANUAL entry group, usage of the random strategy gradually decreased (Fig 5B) and a significant decrease was observed between Days 1, 2, and 6 (*p* < 0.05, Table K in S1 File). In contrast, in the LIFT entry group, usage of the random strategy rapidly decreased between Days 4 and 5 (Fig 5B), and a significant decrease was also detected between Days 1, 2, 4, and 5 (*p* < 0.05, Table K in S1 File). These results indicated that, in the LIFT entry group, a relatively clear change point in the usage of the random strategy was detected between Days 4 and 5, whereas, in the MANUAL entry group, usage of the random strategy gradually decreased between Days 1, 2, and 6, with no apparently clear change point. It was likely that, in the LIFT entry group, after the change point from the random strategy, the usage of the serial and spatial strategy increased in a comparable rate with the random strategy.

### Network analysis

Weiss et al. (2012) [31] demonstrated that the object exploration behaviors of rats could be visualized as structured networks of interconnected nodes. We applied a network analysis to the Barnes maze data and hypothesized that spatial learning in the maze could be described as systematic changes in the network structures of search behaviors.

**Fig 6:**
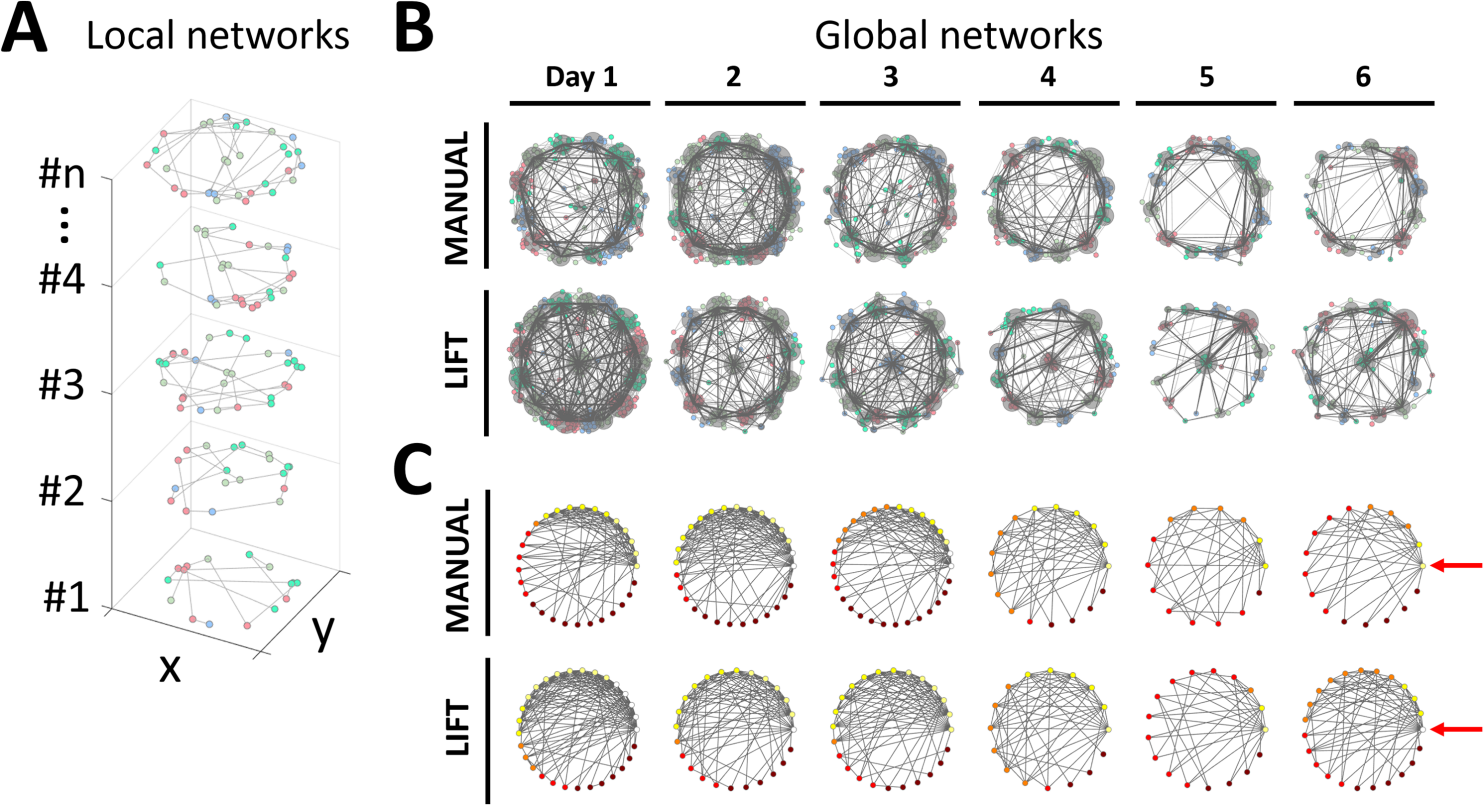
Visualization of temporal changes in the search networks during spatial learning in the MANUAL and LIFT entries. (A) Exmples of local networks from an individual mouse on Day 1 of the LIFT entry. Local networks of mouse #1 to mouse #n generated by the dynamic node generation method are shown in a three-dimentional coordinate system. One local network was generated following a single trial of a particular mouse. The x- and y-axes indicate the plane coordinates, including the arena. The z-axis indicates the sample numbers. The small dots and light gray lines indicate local nodes and links, respectively. One of four colors is assigned to each local node in accordance with a global node associated with the local node (see below). (B) Temporal changes in the global networks in the MANUAL and LIFT entries during spatial learning. Network structures of exploratory behaviors formed by the dynamic node generation method in the MANUAL and LIFT entries are plotted in the upper and lower rows, respectively. Each column corresponds to a single training day. Small colored dots and light gray lines represent nodes and links in the local networks. The local nodes are represented by any one of four colors. Note that this color-coding simply distinguishes geometrically adjacent local nodes belonging to different global nodes using different colors. To describe the global network structure, all local networks of the same entry group on a single training day, such as Fig 6A, were projected on plane coordinates. Dark gray circles and dark gray lines are global nodes and links in global networks, respectively. The magnitude of each global node was determined using logarithmic transformation of the number of local nodes assigned to each global node. A similar procedure was performed to determine global links from sets of local links. The network structure simplified as spatial learning was established. (C) Topological expression of global networks. The layout of rows and columns are the same as in Fig 6B. Global nodes were sorted by rank-order of degree (*M*_*i*_) and plotted on polar coodinates, so that the node with the highest degree was located at 0 degrees while the lowest one was located at 360 degrees. The locations of the global nodes that are most densely linked are highlighted by red arrows. Circles and lines represent global nodes and links, respectively. Each sorted global node is represented by any one of five colors by the magnitude of degree; *white* (*M*_*i*_ ≥ 12), *yellow* (12 > *M*_*i*_ ≥ 9), *orange* (9 > *M*_*i*_ ≥ 6), *red* (6 > *M*_*i*_ ≥ 3), *brown* (3 > *M*_*i*_).

First, the data from the training periods of the MANUAL and LIFT entry groups were subjected to the network analysis, in which the nodes were defined by the dynamic node generation method (see the Materials & Methods). The network structure of the exploratory behaviors of individual mice per trial (local network) was visualized, and then the local networks for each entry group were compiled into global networks for every training day (Fig 6). In Fig 6A and 6B, small colored dots and light gray lines represent nodes and links in the local networks. In Fig 6B, dark gray circles and dark gray lines represent nodes and links in the global networks. It was likely that most local and global nodes were generated near the peripheral holes of the maze, including the target escape hole.

It was apparent that, in both the MANUAL and LIFT entry groups, the network structures simplified as spatial learning was established. In the initial phase of training, each node was densely and uniformly linked to other nodes. In contrast, in the later phase of training, the links connected to the nodes near the target escape hole became strengthened, and links connected indirectly to the target tended to be eliminated (Fig 6B). Thus, systematic changes in the network structures of exploratory behavior during mouse spatial learning have been visualized.

In the strategy analysis (Fig 5B), a statistically significant increase in the use of the spatial strategy was only observed for the LIFT entry group, but not for the MANUAL entry group. This difference was also highlighted in the network analysis: a single direct path between the center global node and the target node emerged in the latter phase of training in the LIFT entry group (Fig 6B). In the LIFT entry group, one global node remained in the center of the arena in the latter phase of learning; in the MANUAL entry group, all global nodes were located at the periphery of the arena. Indeed, when global nodes were sorted by the rank-order of degree within each global network, the overall network structure in the LIFT entry group changed from a crowded to a simplified network compared with the MANUAL entry group (Fig 6C). The generation of a global node in the center of the LIFT entry group could be attributable to the initial immobility of mice in the lift upon entry into the maze. Therefore, we excluded this possibility by removing from the analysis any image sequences in which the mice remained on the lift platform.

**Fig 7:**
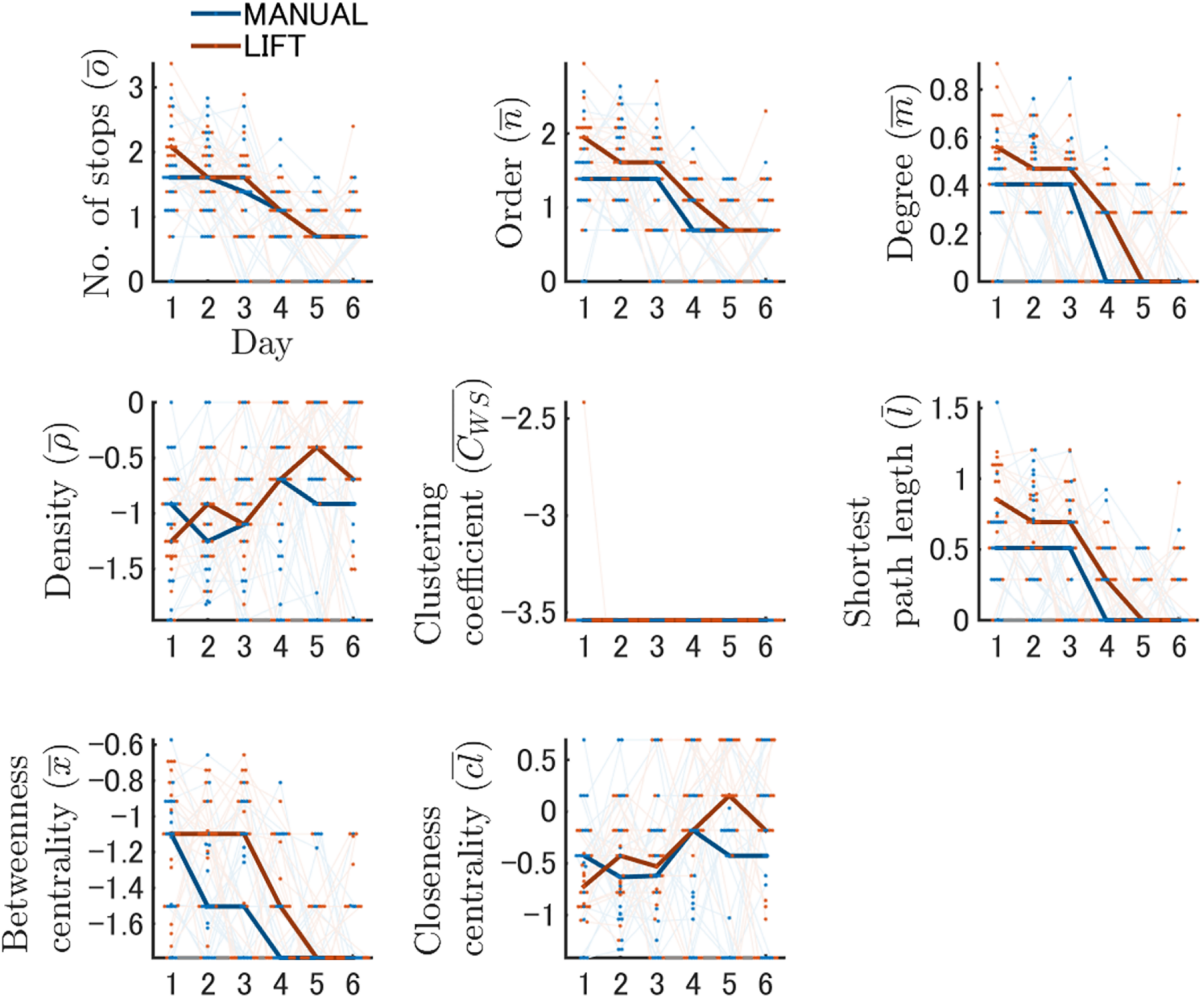
Learning-dependent changes in network features during training. Network features generated by the dynamic node generation method during spatial learning are shown. The results of each network feature on a log scale are described on each individual panel. The vertical and horizontal axes indicate the values of each network feature and training day, respectively. The results of the MANUAL and LIFT entries are shown in *blue* and *red*, respectively. Bold lines represent the changes in the median values in each entry group. Red or blue dots indicate the actual values of an individual mouse in a single day, and the changes across days are depicted by light lines. Simplifying processes of network structures were quantified with temporal changes in network features, indicating that the values of network features changed more apparently in the LIFT entry than in the MANUAL entry.

One strong advantage of the network analysis was that each feature of local networks could be extracted as quantitatively comparable values. The Kruskal–Wallis test for each network feature during training revealed that the components comprising search networks differed between the MANUAL and LIFT entries on all training days except for Day 3 (Fig 7; Tables L and O in S1 File). The number of stops was significantly greater for LIFT entry than for MANUAL entry on Day 1 (χ^2^ (1) = 7.84, *p* < 0.05). The order of networks was also higher for LIFT entry than for MANUAL entry (χ^2^ (1) = 8.64, *p* < 0.05). The degree was greater with LIFT than with MANUAL entry on Day 1 (χ^2^ (1) = 9.84, *p* < 0.05). In contrast, the density on Day 1 was lower for LIFT entry than for MANUAL entry (χ^2^ (1) = 4.35, *p* < 0.05). Interestingly, this relationship was reversed on Day 5 (χ^2^ (1) = 6.14, *p* < 0.05). The shortest path length was higher for LIFT than for MANUAL entry on Day 1 (χ^2^ (1) = 8.86, *p* < 0.05). The betweenness centrality was greater with LIFT than with MANUAL entry on Day 2 (χ^2^ (1) = 6.13, *p* < 0.05).

The Friedman test reported that the change in some features across training differed between the LIFT and MANUAL entry groups (Fig 7; all statistics are shown in Tables M and N in S1 File). The number of stops for LIFT entry was significantly lower on Days 5 and 6 than on Days 1 and 2, in addition to a decrease on Day 4 compared with Day 1 (*p* < 0.05 each, Table N in S1 File). However, for MANUAL entry, such a decrease during training was only observed between Days 1 and 6 (*p* < 0.05, Table N in S1 File). Similar changes were observed in the order, degree, and shortest path length of the networks (*p* < 0.05 each, Table N in S1 File). For MANUAL entry, a significant reduction in the betweenness centrality was only observed between Days 1 and 5 and 6. In contrast, for LIFT entry, a significant decrease in the betweenness centrality was observed between Days 1 and 5 and Days 2 and 5 and 6. The density for LIFT entry was significantly lower on Day 5 compared with Days 1 and 2 (*p* < 0.05 each, Table N in S1 File), as well as being lower on Day 4 compared with Day 1 (*p* < 0.05, Table N in S1 File). Although the closeness centrality in the LIFT entry changed significantly during training (χ^2^ (5) = 11.35, *p* < 0.05, Table M in S1 File), the pairwise comparison found no significant differences between the days (Table N in S1 File). These results indicated that the values of network features changed more noticeably with LIFT entry than with MANUAL entry.

In addition to the network analysis using dynamic nodes, we also conducted a network analysis using static nodes. In this network analysis, a trial point sequence was converted to a sequence of the nearest static nodes (see the Materials & Methods). We observed similar trends in changes in network features between LIFT and MANUAL entry using the static node generation method during the training periods (Fig C in S2 File, Tables O–Q in S1 File).

**Fig 8:**
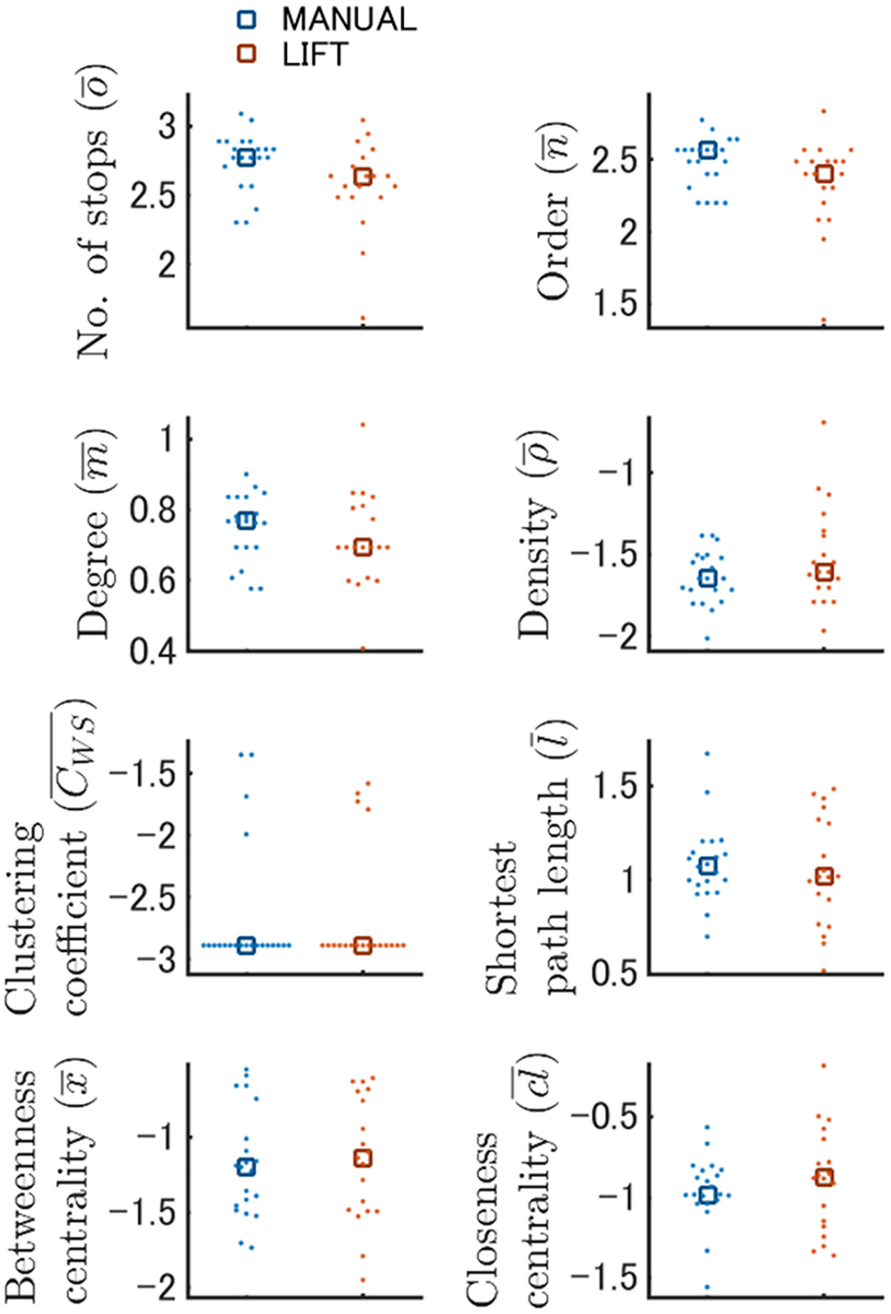
Network features generated by the dynamic node generation method were comparable between the MANUAL and LIFT entries in the probe test. The results of the MANUAL and LIFT entries of the probe tests on a log scale are shown in *blue* and *red*, respectively. Dots and squares indicate the raw and median values, respectively.

Finally, we analyzed the probe test data using network analysis with both the dynamic and static node generation methods (Fig 8; Fig D in S2 File). The network features in both node generation methods were comparable for LIFT and MANUAL entry (Fig 8; Fig D in S2 File). These results indicate that the two mouse groups similarly recalled acquired spatial memories in terms of their search behaviors, even though the two groups might have learned the maze via different systematic changes in exploratory network structures.

### Detecting anomalies in behavioral phenotypes under non-selective muscarinic receptor antagonist administration

To demonstrate the utility of our novel experimental and analytical platform for the spatial learning and memory task, we administered the non-selective muscarinic receptor antagonist scopolamine to the trained mice and analyzed their exploratory behaviors in the probe test. Treatment of animals with a muscarinic receptor blocker before spatial memory retrieval affects the animals’ exploratory behaviors [35–37]. Each mouse was administered either vehicle or scopolamine immediately before the probe test, but not during the training phase. Behavioral phenotypes were clearly different between vehicle (VEH)- and scopolamine (SCOP)-treated mice in the probe test, as described below (S2 Movie), but were indistinguishable during the training phase (data not shown).

In the conventional analysis of the probe test data, the two-way ANOVA showed a significant interaction between treatment and hole (*F*_11,385_ = 2.55, *p* < 0.05, Table R in S1 File). Although the mice of both groups spent significantly more time around the target than around other holes (*p* < 0.05, Table T in S1 File), the SCOP group mice spent significantly longer time around holes near the target compared with the VEH group mice (*p* < 0.05, Fig E in S2 File, Table S in S1 File). Conversely, time spent around the two holes located at –180 and –150 degrees from the target were significantly shorter in the SCOP group than in the VEH group (*p* < 0.05, Fig E in S2 File, Table S in S1 File). These results indicate that scopolamine-treated mice showed better retrieval performance than vehicle-treated mice. Another interpretation is that scopolamine-treated mice persisted in the learned target hole even after they realized the escape box was removed.

When we visualized search networks formed in the probe test, nodes and links in the SCOP group seemed to be more focused around the target compared with those in the VEH group (Fig F in S2 File). Indeed, the Kruskal–Wallis test for each network feature revealed a significant difference between treatments for all features except the number of stops and the clustering coefficient (Figs G and H in S2 File, Table U in S1 File); the order, degree, shortest path length, and betweenness centrality were significantly lower in the SCOP group than in the VEH group (*p* < 0.05, respectively), whereas the density and the closeness centrality were higher in the former than in the latter (*p* < 0.05, respectively).

Therefore, anomalies in behavioral phenotypes induced by scopolamine treatment were detected and visualized by network analysis. Although, in this experiment, scopolamine treatment induced apparent abnormal exploratory behaviors and these changes were detected by conventional analysis, network analysis successfully provided more detailed and quantitative information on animals’ behavioral changes.

## Discussion

### Broad utility of a lift-type task start system for behavioral tasks

For this study, we developed a start-lift and equipped a Barnes maze with it. The subject mouse was raised by the lift and released into the maze automatically, so that it started to navigate the maze smoothly from exactly the same position across trials. In most cases of the Barnes maze test, subject mice are released into the maze manually by experimenters or semi-automatically with a start box [1,3,19,27–29]. Hence, the location of the starting point may fluctuate for each mouse due to inconsistent manual release procedures. In addition, when undesired images of experimenters are captured during manual release, data preprocessing is sometimes required to eliminate the initial image sequence frames. These problems may result in experimental artifacts. Indeed, in the LIFT entry group, the initial mouse start positions were restricted to a narrower area than in the MANUAL entry group (Fig A in S2 File), indicating that more intact data were preserved with the LIFT entry method. Furthermore, more apparent changes in spatial learning were observed for LIFT entry than for MANUAL entry (conventional analysis, Fig 3 and Tables A–C in S1 File; strategy analysis, Fig 5 and Table J in S1 File; network analysis, Figs 6 and 7, Fig C in S2 File, Table M,N,P and Q in S1 File).

A start box is a classic and versatile way to begin behavioral tasks [1,3]. In this method, each mouse is placed inside an opaque or black cylinder and, when the mouse is released into the maze, the cylinder is removed manually or semi-automatically with a pulley. To provide another option for the smooth and stable release of mice, we developed a start-lift and equipped a maze with it. Importantly, the behavioral scores of spatial learning and reference memory analyzed by conventional methods were comparable between the MANUAL and LIFT entry groups. Therefore, the lift-type task start system is a useful option for the Barnes maze apparatus and better suited to combined experiments that use brain imaging and optogenetics. The lift-type task start system can also be applied to other behavioral tasks, such as open field, novel place/object recognition, social interaction, and radial maze tests.

### Network analysis of spatial exploratory behaviors in the spatial learning and memory task

The utility of network analysis for exploration behavior was shown in the analysis of an object recognition test for rats [31]. We hypothesized that learning in a spatial maze could be described as a systematic change in network structures of search behaviors. With network analysis, the exploratory behaviors of mice in the Barnes maze were visualized as interconnected network structures (Fig 6), and various quantitative features of the search network were provided (Fig 7). As spatial learning was established, the network structures became simplified (Fig 6). In the early phase of the training, each node was intricately linked to other nodes in a disorganized manner. In contrast, in the late phase of the training, links connected to nodes near the target escape hole became fixed, and links connected indirectly to the target tended to be eliminated. These trends visualized in the global networks were also supported by temporal changes in the values of the network features (Fig 7). For instance, a reduction in the shortest path length (*l*) indicates the generation of efficient paths connected to key nodes; decreased betweenness centrality (*x*) suggests a reduction in the number of intermediate nodes, whereas increases in density (*ρ*) and closeness centrality (*cl*) mean the formation of dense connections between established nodes.

Surprisingly, robust network structures of exploratory behaviors in the Barnes maze became evident in a short period of time. Weiss et al. (2012) [31] analyzed a spatial exploration test and detected search network structures. However, the task length was relatively long at 20 min per trial and the animals repeatedly explored the same regions of the field in each trial. Conversely, one mouse is subjected to many trials during training and each trial is relatively short in the Barnes maze. In particular, on the final day of training, the average latency time to reach the target was 30 seconds for the LIFT entry group (Fig 3B). These results indicate that network analysis can capture small and rapid changes in animal exploratory behaviors with quantitative values of network components.

Network analysis can potentially be applied to the analysis of various behavioral experiments including other spatial learning and memory tasks, such as the Morris water maze test. We propose that network analysis with the static node generation method would be more stable for the analysis of the Morris water maze data because the dynamic node generation method in the present study generated one node at the stopping coordinates and stopping behavior is less frequently observed in swimming mice. On the other hand, the nodes in the static node generation method were *a priori* generated at the predefined zones in the field (Fig B in S2 File), and animals’ exploratory network can be described as a zone-to-zone transition. We showed that the network features in the network analysis with the static node generation method changed in a learning-dependent manner (Fig D in S2 File). The static node generation method could also be applied to the analysis of the probe test data (Fig D in S2 File). From these results, network analysis with the static node generation method rather than the dynamic one would be more suitable for analysis of the Morris water maze data.

Currently, it is unclear what kinds of external and internal information are critical to determine an animal’s search network structures. The subjective sense of space is mapped by a widespread neural circuit of functionally specialized neuronal cell types located in interconnected brain regions, such as the hippocampus or entorhinal cortex [38]. Place cells in the hippocampus of rodents are one of the most striking examples of a correlation between neuronal activity and complex behavior in mammals [39]. These cells increase their firing rates when the animal visits specific regions of its surroundings, providing a context-dependent map of the environment. The firing pattern of grid cells in the entorhinal cortex provides an intrinsic metric for space [40]. Therefore, place cells supply focal positional information, whereas grid cells supply distance information. Also, self-motion signals are important for animal navigation (“path integration”) and for updating hippocampal location-specific firing. Path-integrative calculation of location is thought to be achieved by head direction cells, which increase their firing rates only when the animal's head points in a specific direction [41,42]. The activity of head direction cells depends on internal cues, such as information from the vestibular system [43]. Therefore, it is likely that, during the maze trials, animals combined intrinsic, path-integrative calculations of location with learned associations of the external environment [44–46]. Thus, we look forward to future studies analyzing how these neural architectures representing an animal’s internal and external spatial information may contribute to the formation, maintenance, and re-mapping of exploratory network structures.

### Relationship between the search strategy and network structure

In the strategy analysis, as spatial learning proceeded, the spatial strategy was increasingly employed and the use of the random strategy became less prevalent (Fig 5). Although it is currently not clear which types of subnetwork may represent different search strategies, it seems that one search strategy contains multiple types of subnetworks. This speculation is derived from the fact that some network features changed independently of the search strategy. For example, a significant reduction in random strategy temporal changes was observed in the LIFT entry group around training Day 4 (Fig 5). However, several network features for the same LIFT entry group, including number of stops, order, degree, and shortest path length in the network of the dynamic node generation method, changed more rapidly: these features changed significantly before Day 4 (Fig 7 and Table N in S1 File), indicating that changes in network features do not perfectly correlate with changes in the employment of search strategies.

It is also noted that we observed several time points with significant differences in network features but not in search strategies between the MANUAL and LIFT entry groups. For instance, on Training Day 1, the proportion of random strategy use was the highest in both the MANUAL and LIFT entry groups (Fig 5 and Table I in S1 File), whereas, in the network analysis, we found significant differences between the two groups for the scores of order, degree, density and shortest path length (Fig 7 and Table L in S1 File). Furthermore, the density scores significantly differed between the groups on Day 5 compared with Training Days 5 and 6 (Fig 7 and Table L in S1 File), despite similar employment of each strategy between the two entry groups (Fig 5 and Table I in S1 File). Our network analysis findings were similar using the static node generation method. These results indicate the possibility that one particular strategy may contain different sets of subnetwork structures.

In the strategy analysis, the definitions of each strategy are determined by experimenters, and it is unclear whether the current criteria are really appropriate. Furthermore, animals might use different kinds of search strategies that were not included in the analysis. In contrast, in the network analysis, no network structures are predetermined and all data analyses are conducted automatically by the program. Therefore, network analysis allows us to quantitatively and non-objectively investigate animals’ exploratory behaviors. However, it is currently unclear how each value of the network features corresponds to animals’ internal brain activities. Real-time brain activity monitoring and/or its interventions will contribute to a deeper understanding of brain activities and neural circuit mechanism regulating animals’ exploratory network structures.

### Utility of network analysis for detecting behavioral anomalies

We believe that network analysis can detect minute but significant changes in animals’ exploratory behaviors caused by genetic or pharmaceutical manipulation of neural circuits. For example, in this study, we showed that behavioral anomalies caused by administration of the non-selective muscarinic receptor antagonist scopolamine in the probe test of the Barnes maze were clearly highlighted with network analysis (Figs E–H in S2 File, Tables R–U in S1 File). Conventional analysis indicated that both VEH and SCOP treatment groups intensely searched regions near the target holes, but not regions far from the targets (Fig E in S2 File), indicating that spatial reference memory was recalled in both groups, although this tendency was more strongly observed in the SCOP group mice. The network analysis also well characterized this spatial learning-dependent animal search behavior. For example, in the local/global network structures, many local nodes were generated around the target holes and only a limited number of nodes was generated around non-target holes. Again, this feature was more apparent in the SCOP treatment group (Fig F in S2 File). Therefore, spatial learning-dependent typical exploratory behaviors were well described in the network analysis, as well as in the conventional analysis.

Furthermore, network analysis can provide more detailed information on behavioral characteristics. For instance, conventional analysis does not provide any information on the transitional patterns between visited spots. In contrast, network analysis enables us to quantitatively analyze how animals explored the maze with specific spatiotemporal patterns. When we compared the search network structures in the probe test between the VEH and SCOP treatment groups, we found that the SCOP group mice exhibited noticeable transitions between the limited nodes near the target, whereas in the VEH group, the transitions between the nodes were relatively uniform across the field (Fig F in S2 File). These fine behavioral changes were quantitatively detected as significant differences in the network feature values between the two groups (Figs G–H in S2 File). Thus, network analysis would shed light on novel behavioral phenotypes that were not described by the conventional data analyses.

We observed that scopolamine treatment induced a focal search strategy in the Barnes maze probe test. Similarly, previous studies reported that scopolamine-treated mice in the probe test of the Morris water maze displayed a focal search strategy [35,36]. However, impaired spatial memory recall was also observed. In our Barnes maze result, scopolamine-treated mice stayed longer near the target hole and therefore showed better retrieval performance than vehicle-treated mice. This discrepancy might be due to the different topographic features between the Morris water maze and Barnes maze. In the Barnes maze, the goal positions are located in the peripheral region of the field, whereas in the water maze, the goal is located somewhat closer to the center of the maze. Therefore, when the mice recognize that the goal no longer exists in the original target location, subsequent searching areas might be different between the Barnes and Morris water maze tests. For example, in the Barnes maze, all possible holes are placed peripherally and all of these holes can be seen by the mouse. However, in the water maze, if mice cannot find the escape platform, they have to search more broader regions of the maze because there are no visible indications of the goal similar to the visible holes of the Barnes maze. Thus, even if mice adopt fundamentally similar search strategies, their behavioral outcomes and calculated scores in the probe test could be different between the two tests.

A number of studies reported that retrieval from stored memory and encoding of the current state of the environment are associated with information flows from CA3 to CA1 and from the entorhinal cortex (EC) to CA1, respectively [47,48]. Each information flow is represented as burst firing of CA1 cells at a specific phase within the theta cycle [47–50]. The retrieval related signals from CA3 are strengthened by scopolamine treatment because acetylcholine selectively suppresses the signal from CA3 but not from the EC [47,51]. In fact, scopolamine treatment in a well-habituated environment shifts the preferred firing phase of the CA1 cell population toward a theta trough corresponding to the phase where the CA3 send the retrieval-related signal to the CA1 [47,51].

### Detailed exploratory behaviors as a drug-screening platform for the early treatment of neural disorders

A high sensitivity assay system to identify cognitive impairment in model animals and to evaluate therapeutic effects is greatly needed for basic research and drug discovery in the study of neural diseases [6,7]. For example, the development of an assay system for AD research that can identify cognitive deficits with sufficient sensitivity would be advantageous. After the amyloid plaque deposition becomes apparent, drugs do not work efficiently. For instance, γ-secretase inhibitor treatment in the Tg2676 AD model mice from 4 to 7 months of age resulted in a greater decrease in amyloid beta (Aβ) levels than treatment initiation after drastic increases in amyloid plaque deposition; the same treatment initiated at 7 to 10 months was less effective [52]. Therefore, it is reasonable to seek and screen drugs that have considerable therapeutic potential especially in the early phase of AD. However, most AD model mouse strains do not show statistically significant deficits at a young age, such as 4 months, with the standard Morris water maze or Barnes maze tests [53–56]. Therefore, many groups have tried to improve the maze protocols to detect subtle deficits in learning and memory [6,7]. In combination with these efforts, the fine description of an animal’s exploratory behavior achieved by network analysis may be a potential platform for drug screening in neural disorders, such as AD, and may contribute to the development of new therapeutic strategies.

## Supporting information

S1 File Supplementary tables. **S1** File contains tables A to U.
(PDF)

S2 File Supplementary figures. **S2** File contains figures A to H.
(PDF)

S1 Movie The dynamic node generation process in network analysis. The dynamic node generation process for a local network of a representative mouse case (#52, the LIFT group, the first trial of Day 1) is shown in the movie. The first part of the movie shows the generation of stopping coordinates from the mouse trajectory, and the second part shows the generation of nodes and links from the stopping coordinates. In the generation of stopping coordinates, the current mouse position and moving trajectory are indicated by a red plus symbol and black lines, respectively. Stopping coordinates and transitions between them are indicated by cyan-filled circles and cyan dotted lines, respectively. The stopping coordinates and transitions are similarly illustrated for the generation of nodes. First, the program selected the coordinates of a stopping point and then integrated these coordinates into the nearest node when a distance of less than 20 pixels between the stop and node was reached. If a stop was not integrated into any node under this criterion, the stop was treated as a new node. Second, the coordinates of all nodes were calculated, and each node was represented by the centroid of its constituent stops. Third, the program determined whether all stopping coordinates were integrated into a node. If not, the processing returned to the first step. Eventually, each node had unique coordinates, and a history of transitions between nodes was also determined per trial; each transition was treated as an undirected link between any two nodes (see the Material and Methods for details).
(MP4)

S2 Movie Exploratory behaviors of the VEH and SCOP groups in the probe test. Representative movies of VEH-treated mice (VEH #3 and VEH #23) and SCOP-treated mice (SCOP #15 and SCOP #16) are shown. The trajectories of mice are shown with colored lines and the target locations are indicated by arrows.
(MP4)
