# Supplementary material for: Network analysis of exploratory behaviors of mice in a spatial learning and memory task
Source: PLoS One. 2017 Jul 10;12(7):e0180789. doi: 10.1371/journal.pone.0180789 (PMC5507260; doi:10.1371/journal.pone.0180789)
Supplement: S1 File — contains tables A to U. (PDF) [file pone.0180789.s001.pdf]

Table A. Analysis of variance (ANOVA) for conventional features of the entries across training days. The columns indicate feature, source of variation, sum of squares (SS), degree of freedom (df), mean square (MS), F value, *p* value, and alternative hypothesis (H_1). An asterisk indicates that an alternative hypothesis was true.

Feature	Source	SS	df	MS	F	*p*	H_1
No. of errors	Main effect: Entry	11.06	1	11.06	0.28	0.60	
	Error: S(Entry)	1,507.51	38	39.67			
	Main effect: Day	1,720.64	5	344.13	19.06	0.00	*
	Interaction: Entry × Day	424.06	5	84.81	4.70	0.00	*
	Error: Day × S(Entry)	3,429.96	190	18.05			
Latency	Main effect: Entry	1,286.11	1	1,286.11	0.24	0.63	
	Error: S(Entry)	206,273.61	38	5,428.25			
	Main effect: Day	46,958.75	5	9,391.75	9.63	0.00	*
	Interaction: Entry × Day	20,512.04	5	4,102.41	4.21	0.00	*
	Error: Day × S(Entry)	185,289.33	190	975.21			
Travel distance	Main effect: Entry	2,768,999.56	1	2,768,999.56	3.01	0.09	
	Error: S(Entry)	35,002,939.14	38	921,129.98			
	Main effect: Day	54,652,073.01	5	10,930,414.60	22.55	0.00	*
	Interaction: Entry × Day	15,684,835.96	5	3,136,967.19	6.47	0.00	*
	Error: Day × S(Entry)	92,109,257.05	190	484,785.56			

Table B. Multiple comparisons by Tukey's honest significant difference (HSD) test for the effect of entry mode on Day 1. The columns indicate feature, day, degree of freedom, *t* value, alternative hypothesis, and Pearson's *R*. An asterisk indicates that an alternative hypothesis was true.

**Feature**	**Day**	**df**	***t***	**H_1**	***R***
No. of errors	1	38	2.97	*	0.43
Latency	1	38	3.00	*	0.44
Travel distance	1	38	3.87	*	0.53

Table C. Multiple comparisons between days within each conventional feature. Each sub-table shows the pairwise comparisons by Tukey's HSD test. An asterisk with an effect size (Pearson's R) is used to indicate a significant difference in the values of a conventional feature between any 2 days.

**Entry**	**Feature**		**1**	**2**	**3**	**4**	**5**	**6**
MANUAL	No. of errors	1						*(0.63)
		2				*(0.52)	*(0.73)	*(0.73)
		3						
		4						
		5						
		6						
	Latency	1						
		2					*(0.79)	*(0.81)
		3						*(0.35)
		4						
		5						
		6						
	Travel distance	1					*(0.59)	*(0.66)
		2				*(0.45)	*(0.79)	*(0.81)
		3						
		4						
		5						
		6						
LIFT	No. of errors	1		*(0.64)	*(0.60)	*(0.82)	*(0.84)	*(0.82)
		2					*(0.76)	
		3					*(0.69)	
		4						
		5						
		6						
	Latency	1		*(0.61)	*(0.67)	*(0.81)	*(0.82)	*(0.84)
		2						
		3						
		4						
		5						
		6						
	Travel distance	1		*(0.69)	*(0.69)	*(0.82)	*(0.86)	*(0.83)
		2					*(0.81)	
		3					*(0.72)	
		4						
		5						
		6						

Table D. ANOVA for the time spent around each hole between entries. The columns indicate feature, source of variation, sum of squares, degree of freedom, mean square, F value, *p* value, and alternative hypothesis. An asterisk indicates that an alternative hypothesis was true.

Feature	Source	SS	df	MS	F	*p*	H_1
No. of visits	Main effect: Entry	478.80	1	478.80	1.51	0.23	
	Error: S(Entry)	12,079.17	38	317.87			
	Main effect: Angle	414,119.32	11	37,647.21	39.48	0.00	*
	Interaction: Entry × Angle	12,413.32	11	1,128.48	1.18	0.00	*
	Error: Angle × S(Entry)	398,609.81	418	953.61			

Table E. Multiple comparisons of time spent around each hole in the effect of entry as determined by Tukey's HSD test. The columns indicate feature, angle, degree of freedom, *t* value, alternative hypothesis, and Pearson's *R*. An asterisk indicates that an alternative hypothesis was true.

**Feature**	**Angle**	**df**	***t***	**H_1**	***R***
Time spent around each hole (probe test)	Target	38	1.10	*	0.18

Table F. Multiple comparisons between holes in the probe test within each conventional feature. Each sub-table shows pairwise comparisons by Tukey's HSD test. An asterisk with an effect size (Pearson's *R*) is used to indicate a significant difference in the values of a conventional feature between any 2 days.

**Entry**		**−180**	**−150**	**−120**	**−90**	**−60**	**−30**	**0**	**30**	**60**	**90**	**120**	**150**
	−180							*(0.71)					
	−150							*(0.73)					
	−120							*(0.75)					
	−90							*(0.66)					
	−60							*(0.70)					
	−30							*(0.74)					
MANUAL	0								*(0.72)	*(0.76)	*(0.71)	*(0.74)	*(0.76)
	30												
	60												
	90												
	120												
	150												
	−180							*(0.70)					
	−150							*(0.82)					
	−120							*(0.83)					
	−90							*(0.79)					
	−60							*(0.81)					
	−30							*(0.75)					
LIFT	0								*(0.81)	*(0.82)	*(0.79)	*(0.82)	*(0.79)
	30												
	60												
	90												
	120												
	150												

Table G. Kruskal–Wallis test for the usage of search strategies between the MANUAL and LIFT entry groups each day. The columns indicate training day, search strategy, degree of freedom, chi-square value (χ^2), *p* value, alternative hypothesis, and test number. An asterisk indicates that an alternative hypothesis was true. No significant differences were observed at the 5% level.

Day	Strategy	df	χ^2	*p*	H_1	Test
1	Random	1	1.30	0.25		1
	Serial	1	0.41	0.52		2
	Spatial	1	0.56	0.45		3
2	Random	1	0.11	0.74		4
	Serial	1	0.10	0.75		5
	Spatial	1	0.04	0.84		6
3	Random	1	0.90	0.34		7
	Serial	1	0.24	0.62		8
	Spatial	1	0.11	0.74		9
4	Random	1	1.14	0.29		10
	Serial	1	3.26	0.07		11
	Spatial	1	0.26	0.61		12
5	Random	1	0.00	0.97		13
	Serial	1	0.46	0.50		14
	Spatial	1	1.24	0.27		15
6	Random	1	0.34	0.56		16
	Serial	1	0.22	0.64		17
	Spatial	1	0.02	0.90		18

Table H. Friedman test for strategy usage within a single day for every entry. The columns indicate entry mode, training day, degree of freedom, chi-square value, *p* value, alternative hypothesis, and test number. An asterisk indicates that an alternative hypothesis was true.

**Entry**	**Day**	**df**	χ^2	p	H_1	**Test**
MANUAL	1	2	22.44	0.00	*	1
	2	2	23.78	0.00	*	2
	3	2	8.20	0.02	*	3
	4	2	4.78	0.09		4
	5	2	1.19	0.55		5
	6	2	0.35	0.84		6
LIFT	1	2	23.64	0.00	*	7
	2	2	13.69	0.00	*	8
	3	2	10.70	0.00	*	9
	4	2	12.11	0.00	*	10
	5	2	0.60	0.74		11
	6	2	0.76	0.68		12

Table I. Multiple comparisons of strategy usage. The table shows pairwise comparisons as determined using the sign test adjusted with the Ryan procedure for each test in which a significant difference was indicated in S8 Table. An asterisk indicates that an alternative hypothesis was true.

**Entry**	**Day**		**Random**	**Serial**	**Spatial**
MANUAL	1	Random		*	*
		Serial			
		Spatial			
	2	Random		*	*
		Serial			
		Spatial			
	3	Random		*	
		Serial			
		Spatial			
LIFT	1	Random		*	*
		Serial			
		Spatial			
	2	Random		*	*
		Serial			
		Spatial			
	3	Random		*	*
		Serial			
		Spatial			
	4	Random		*	
		Serial			
		Spatial			

Table J. Friedman test for strategy usage changes by entry type across days. The columns indicate entry mode, strategy, degree of freedom, chi-square value, *p* value, alternative hypothesis, and test number. An asterisk indicates that an alternative hypothesis was true.

**Entry**	**Strategy**	**df**	χ^2	***p***	**H_1**	**Test**
MANUAL	Random	5	23.98	0.00	*	1
	Serial	5	19.94	0.00	*	2
	Spatial	5	8.22	0.14		3
LIFT	Random	5	31.20	0.00	*	4
	Serial	5	18.90	0.00	*	5
	Spatial	5	18.41	0.00	*	6

Table K. Multiple comparisons of temporal changes for each strategy. The table shows pairwise comparisons as determined using the sign test adjusted with the Ryan procedure for each test in which a significant difference was indicated in S10 Table. An asterisk indicates that an alternative hypothesis was true.

**Entry**	**Strategy**		**1**	**2**	**3**	**4**	**5**	**6**
MANUAL	Random	1						*
		2						*
		3						
		4						
		5						
		6						
	Serial	1					*	
		2						
		3						
		4						
		5						
		6						
LIFT	Random	1					*	*
		2					*	
		3						
		4					*	
		5						
		6						
	Serial	1						
		2						
		3						
		4						
		5						
		6						
	Spatial	1						
		2						
		3						
		4						

5
6

Table L. Kruskal–Wallis test for network features of the dynamic node generation method between the MANUAL and LIFT entries per day. The columns indicate node generation method, feature, day, degree of freedom, chi-square value, p value, alternative hypothesis, and test number. An asterisk indicates that an alternative hypothesis was true.

Node generation method	Feature	Day	df	χ^2	p	H_1	Test
Dynamic	$\bar{o}$	1	1	7.84	0.01	*	1
		2	1	0.04	0.84		2
		3	1	0.36	0.55		3
		4	1	2.67	0.10		4
		5	1	0.08	0.77		5
		6	1	2.26	0.13		6
	$\bar{n}$	1	1	8.64	0.00	*	7
		2	1	0.07	0.80		8
		3	1	1.02	0.31		9
		4	1	2.95	0.09		10
		5	1	0.60	0.44		11
		6	1	2.27	0.13		12
	$\bar{m}$	1	1	9.84	0.00	*	13
		2	1	0.23	0.63		14
		3	1	0.76	0.38		15
		4	1	3.79	0.05		16
		5	1	0.60	0.44		17
		6	1	2.27	0.13		18
	$\bar{\rho}$	1	1	4.35	0.04	*	19
		2	1	0.05	0.83		20
		3	1	0.63	0.43		21
		4	1	1.23	0.27		22
		5	1	6.14	0.01	*	23
		6	1	0.46	0.50		24
	$\overline{C_{WS}}$	1	1	2.27	0.13		25
		2	1	2.27	0.13		26
		3	1	0.90	0.34		27
		4	1	N/A	N/A	N/A	28
		5	1	N/A	N/A	N/A	29
		6	1	N/A	N/A	N/A	30

	1	1	8.86	0.00	*	31
	2	1	0.23	0.63		32
$\bar{l}$	3	1	1.13	0.29		33
	4	1	2.71	0.10		34
	5	1	0.60	0.44		35
	6	1	2.35	0.12		36
	1	1	0.28	0.60		37
	2	1	6.13	0.01	*	38
$\bar{x}$	3	1	1.29	0.26		39
	4	1	0.12	0.73		40
	5	1	0.09	0.77		41
	6	1	2.48	0.12		42
	1	1	2.99	0.08		43
	2	1	0.54	0.46		44
$\overline{cl}$	3	1	0.02	0.89		45
	4	1	1.13	0.29		46
	5	1	3.75	0.05		47
	6	1	0.05	0.82		48

Table M. Friedman test for changes in network features of the dynamic node generation method across days within entry. The columns indicate entry mode, node generation method, network feature, degree of freedom, chi-square value, *p* value, alternative hypothesis, and test number. An asterisk indicates that an alternative hypothesis was true.

Entry	Node generation method	Feature	df	χ^2	*p*	H_1	Test
MANUAL	Dynamic	$\bar{o}$	5	27.98	0.00	*	1
		$\bar{n}$	5	26.87	0.00	*	2
		$\bar{m}$	5	27.37	0.00	*	3
		$\bar{\rho}$	5	4.16	0.53		4
		$\overline{C_{\mathrm{WS}}}$	5	5.00	0.42		5
		$\bar{l}$	5	26.42	0.00	*	6
		$\bar{x}$	5	27.30	0.00	*	7
		$\overline{cl}$	5	0.58	0.99		8
LIFT	Dynamic	$\bar{o}$	5	46.32	0.00	*	16
		$\bar{n}$	5	46.79	0.00	*	17
		$\bar{m}$	5	45.02	0.00	*	18
		$\bar{\rho}$	5	34.39	0.00	*	19
		$\overline{C_{\mathrm{WS}}}$	5	8.82	0.12		20
		$\bar{l}$	5	46.44	0.00	*	21
		$\bar{x}$	5	31.86	0.00	*	22
		$\overline{cl}$	5	11.35	0.04	*	23

Table N. Multiple comparisons between days within each network feature of the dynamic node generation method. The table shows pairwise comparisons as determined using the sign test adjusted with the Ryan procedure for each test in which a significant difference was indicated in S13 Table. If there was a significant difference in the value of a network feature between any 2 days, an asterisk marks the corresponding cell.

Entry	Node generation method	Feature		1	2	3	4	5	6
MANUAL	Dynamic	$\bar{o}$	1						*
			2						
			3						
			4						
			5						
			6						
		$\bar{n}$	1						*
			2						
			3						
			4						
			5						
			6						
		$\bar{m}$	1						*
			2						
			3						
			4						
			5						
			6						
		$\bar{l}$	1						*
			2						
			3						
			4						
			5						
			6						
		$\bar{x}$	1					*	*
			2						
			3						
			4						

			5			
			6			
LIFT	Dynamic	$\bar{o}$	1	*	*	*
			2		*	*
			3			
			4			
			5			
			6			
		$\bar{n}$	1	*	*	*
			2		*	*
			3			
			4			
			5			
			6			
		$\bar{m}$	1	*	*	*
			2		*	*
			3			
			4			
			5			
			6			
		$\bar{\rho}$	1	*	*	
			2		*	
			3			
			4			
			5			
			6			
		$\bar{l}$	1	*	*	*
			2		*	*
			3			
			4			
			5			
			6			
		$\bar{x}$	1		*	
			2		*	*
			3			
			4			
			5			
			6			

$\bar{c}l$	1
	2
	3
	4
	5
	6

Table O. Kruskal–Wallis test for the network features of the static node generation method between the MANUAL and LIFT entry groups per day. The columns indicate the node generation method, feature, day, degree of freedom, chi-square value, p value, alternative hypothesis, and test number. An asterisk indicates that an alternative hypothesis was true.

Node generation method	Feature	Day	df	χ^2	p	H_1	Test
Static	$\bar{n}$	1	1	12.26	0.00	*	49
		2	1	1.57	0.21		50
		3	1	1.82	0.18		51
		4	1	3.37	0.07		52
		5	1	1.61	0.20		53
		6	1	5.51	0.02	*	54
	$\bar{m}$	1	1	15.31	0.00	*	55
		2	1	0.33	0.57		56
		3	1	1.92	0.17		57
		4	1	1.40	0.24		58
		5	1	0.09	0.76		59
		6	1	3.58	0.06		60
	$\bar{\rho}$	1	1	9.06	0.00	*	61
		2	1	0.88	0.35		62
		3	1	1.85	0.17		63
		4	1	2.89	0.09		64
		5	1	0.56	0.45		65
		6	1	2.87	0.09		66
	$\overline{C_{\mathrm{WS}}}$	1	1	0.47	0.50		67
		2	1	1.86	0.17		68
		3	1	N/A	N/A	N/A	69
		4	1	N/A	N/A	N/A	70
		5	1	N/A	N/A	N/A	71
		6	1	N/A	N/A	N/A	72
	$\bar{l}$	1	1	1.59	0.21		73
		2	1	0.17	0.68		74
		3	1	0.85	0.36		75
		4	1	4.59	0.03	*	76
		5	1	2.79	0.09		77
		6	1	4.97	0.03	*	78

$\bar{x}$	1	1	10.14	0.00	*	79
	2	1	0.17	0.68		80
	3	1	0.52	0.47		81
	4	1	5.65	0.02	*	82
	5	1	2.27	0.13		83
	6	1	2.32	0.13		84
$\overline{cl}$	1	1	7.94	0.00	*	85
	2	1	1.12	0.29		86
	3	1	1.49	0.22		87
	4	1	3.45	0.06		88
	5	1	0.44	0.51		89
	6	1	2.40	0.12		90

Table P. Friedman test for network features changes in the static node generation method across days by entry type. The columns indicate entry mode, node generation method, network feature, degree of freedom, chi-square value, *p* value, alternative hypothesis, and test number. An asterisk indicates that an alternative hypothesis was true.

Entry	Node generation method	Feature	df	χ^2	*p*	H_1	Test
		$\bar{n}$	5	24.48	0.00	*	9
		$\bar{m}$	5	11.95	0.04	*	10
		$\bar{\rho}$	5	18.34	0.00	*	11
MANUAL	Static	$\overline{C_{\mathrm{WS}}}$	5	7.00	0.22		12
		$\bar{l}$	5	22.67	0.00	*	13
		$\bar{x}$	5	7.16	0.21		14
		$\overline{cl}$	5	21.38	0.00	*	15
		$\bar{n}$	5	34.87	0.00	*	24
		$\bar{m}$	5	35.98	0.00	*	25
		$\bar{\rho}$	5	29.34	0.00	*	26
LIFT	Static	$\overline{C_{\mathrm{WS}}}$	5	10.00	0.08		27
		$\bar{l}$	5	9.26	0.10		28
		$\bar{x}$	5	31.15	0.00	*	29
		$\overline{cl}$	5	29.00	0.00	*	30

Table Q. Multiple comparisons between days within each network feature of the static node generation method. The table shows pairwise comparisons as determined using the sign test adjusted with the Ryan procedure for each test in which a significant difference was indicated in S16 Table. If there was a significant difference in the value of a network feature between any 2 days, an asterisk marks the corresponding cell.

Entry	Node generation method	Feature		1	2	3	4	5	6
MANUAL	Static	$\bar{n}$	1						
			2					*	*
			3						
			4						
			5						
			6						
		$\bar{m}$	1						
			2						*
			3						
			4						
			5						
			6						
		$\bar{\rho}$	1						
			2						*
			3						
			4						
			5						
			6						
		$\bar{l}$	1						
			2						
			3						
			4						
			5						
			6						
		$\overline{cl}$	1						
			2						
			3						
			4						
			5						

			6					
LIFT	Static	$\bar{n}$	1				*	
			2				*	
			3				*	
			4				*	
			5					*
			6					
		$\bar{m}$	1	*	*	*	*	*
			2					
			3					
			4					
			5					
			6					
		$\bar{\rho}$	1				*	*
			2					
			3					
			4					
			5					
			6					
		$\bar{x}$	1	*	*	*	*	*
			2					
			3					
			4					
			5					
			6					
		$\overline{cl}$	1				*	*
			2					
			3					
			4					
			5					
			6					

Table. R ANOVA for the time spent around each hole between the VEH and SCOP groups in the probe test. The columns indicate feature, source of variation, sum of squares, degree of freedom, mean square, F value, *p* value, and alternative hypothesis. An asterisk indicates that an alternative hypothesis was true.

**Feature**	**Source**	**SS**	**df**	**MS**	**F**	***p***	**H_1**
No. of visits	Main effect: Treatment	5,148.69	1	5,148.69	5.54	0.02	*
	Error: S(Treatment)	32,540.22	35	929.72			
	Main effect: Angle	1983,479.60	11	180,316.33	52.49	0.00	*
	Interaction: Treatment × Angle	96284.32	11	8,753.12	2.55	0.00	*
	Error: Angle × S(Treatment)	1322,656.67	385	3,435.47			

Table S. Multiple comparisons of time spent around each hole in the effect of treatment as determined by Tukey's HSD test. The columns indicate feature, angle, degree of freedom, *t* value, alternative hypothesis, and Pearson's *R*. An asterisk indicates that an alternative hypothesis was true.

**Feature**	**Angle (°)**	**df**	***t***	**H_1**	***R***
Time spent around each hole (probe test)	–180	35	3.50	*	0.51
	–150	35	3.77	*	0.54
	Target	35	1.33	*	0.22
	30	35	1.83	*	0.30

Table T. Multiple comparisons between holes in the probe test within each treatment. Each sub-table shows pairwise comparisons by Tukey's HSD test. An asterisk with an effect size (Pearson's *R*) indicates a significant difference in the values of a conventional feature between any two holes.

Treatment		−180	−150	−120	−90	−60	−30	0	30	60	90	120	150
	−180						*(0.55)	*(0.80)					
	−150						*(0.59)	*(0.81)					
	−120						*(0.69)	*(0.86)	*(0.79)				
	−90						*(0.65)	*(0.85)	*(0.76)				
	−60						*(0.69)	*(0.87)	*(0.79)				
VEH	−30							*(0.83)		*(0.70)	*(0.71)	*(0.73)	*(0.69)
	0								*(0.69)	*(0.88)	*(0.89)	*(0.89)	*(0.87)
	30									*(0.83)	*(0.82)	*(0.83)	*(0.77)
	60												
	90												
	120												
	150												
	−180						*(0.87)	*(0.90)	*(0.78)				
	−150						*(0.86)	*(0.89)	*(0.79)				
	−120						*(0.87)	*(0.90)	*(0.81)				
	−90						*(0.79)	*(0.84)	*(0.71)				
	−60						*(0.73)	*(0.83)	*(0.67)				
SCOP	−30							*(0.75)		*(0.79)	*(0.80)	*(0.83)	*(0.89)
	0								*(0.80)	*(0.88)	*(0.87)	*(0.88)	*(0.90)
	30									*(0.75)	*(0.73)	*(0.77)	*(0.79)
	60												
	90												
	120												
	150												

Table U. Kruskal–Wallis test for the network features of both the dynamic and static node generation methods between the VEH and SCOP entry groups in the probe test. The columns indicate the node generation method, feature, degree of freedom, chi-square value, p value, alternative hypothesis, and test number. An asterisk indicates that an alternative hypothesis was true.

Node generation method	Feature	df	χ^2	p	H_1	Test
Dynamic	$\bar{o}$	1	0.00	0.95		1
	$\bar{n}$	1	15.95	0.00	*	2
	$\bar{m}$	1	15.95	0.00	*	3
	$\bar{\rho}$	1	15.95	0.00	*	4
	$\overline{C_{\mathrm{WS}}}$	1	N/A	N/A		5
	$\bar{l}$	1	15.95	0.00	*	6
	$\bar{x}$	1	15.95	0.00	*	7
	$\overline{cl}$	1	15.95	0.00	*	8
Static	$\bar{n}$	1	6.37	0.01	*	9
	$\bar{m}$	1	6.06	0.01	*	10
	$\bar{\rho}$	1	0.12	0.73		11
	$\overline{C_{\mathrm{WS}}}$	1	0.04	0.84		12
	$\bar{l}$	1	5.62	0.02	*	13
	$\bar{x}$	1	0.07	0.78		14
	$\overline{cl}$	1	7.48	0.01	*	15
