# Supplementary material for: Network analysis of exploratory behaviors of mice in a spatial learning and memory task
Source: PLoS One. 2017 Jul 10;12(7):e0180789. doi: 10.1371/journal.pone.0180789 (PMC5507260; doi:10.1371/journal.pone.0180789)
Supplement: S2 File — contains figures A to H. (PDF) [file pone.0180789.s002.pdf]

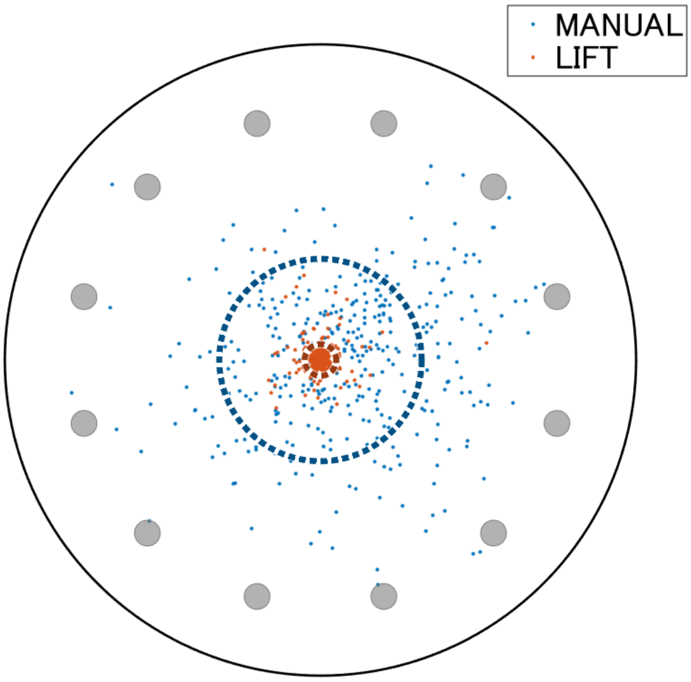


**Figure A. Distribution of the initial positions of each mouse in the MANUAL- and LIFT-entry groups.** The large circle and small gray circles represent the arena and the holes, respectively. The initial mouse positions after data preprocessing are shown in *blue* for the MANUAL entry trials and in *red* for the LIFT entry trials, respectively. The mean distance from the center of the arena in each entry is represented by the blue and red dashed line circle, respectively. In the LIFT entry trials, the initial mouse positions were covered a more restricted area than in the MANUAL entry trials.

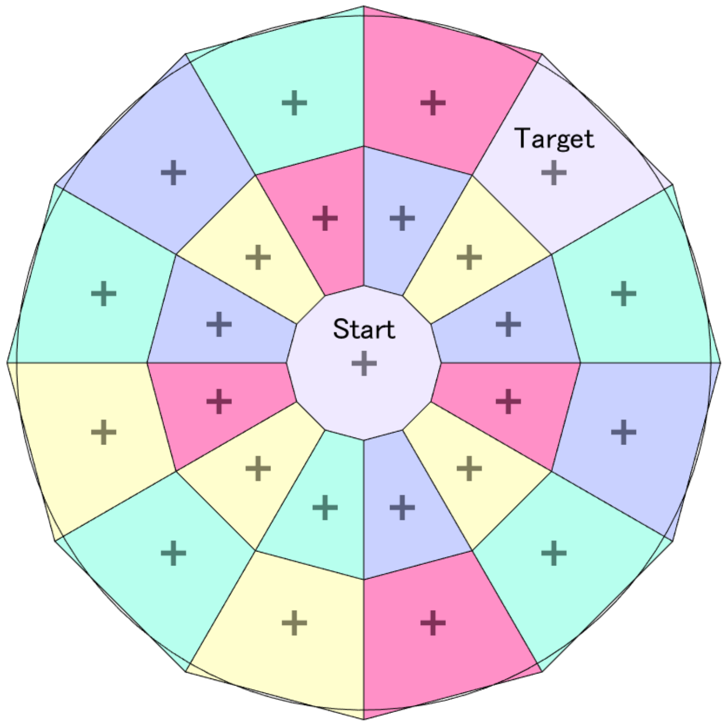


**Figure B. Analytical zones on the maze for the static node generation.** The large outside circle indicates the arena and the small plus marker (“+”) represents the static nodes. A total of 25 static nodes were uniformly scattered across the arena. The surrounding area of each node was designated as the specific zone of the node. Every static node has its own unique static zone (see main text). Each zone is color-coded by any 1 of 5 colors. The coloring simply differentiates adjacent zones. The two light violet zones represent the start and target zones, respectively. “Target” means the location of the target hole after the coordinate transformation (see the Materials & Methods).

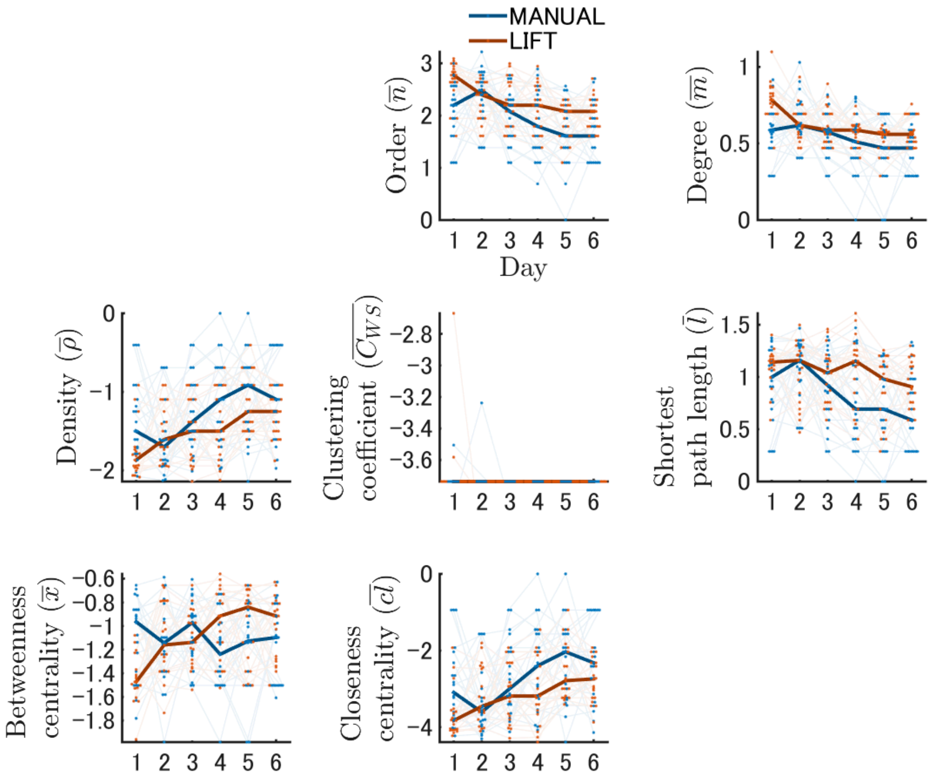


**Figure C. Learning-dependent changes of network features in the network analysis with the static node generation method.** Network features generated by the static node generation method during spatial learning are shown. The results of each network feature on a log scale are described in the individual panels. The vertical and horizontal axes indicate the values of each network feature and training day, respectively. The results of the MANUAL and LIFT entries are shown in *blue* and *red*, respectively. The bold lines represent the changes in the median values for each entry. Red or blue dots indicate the actual values of a mouse each day, and the changes across days are depicted by light lines.

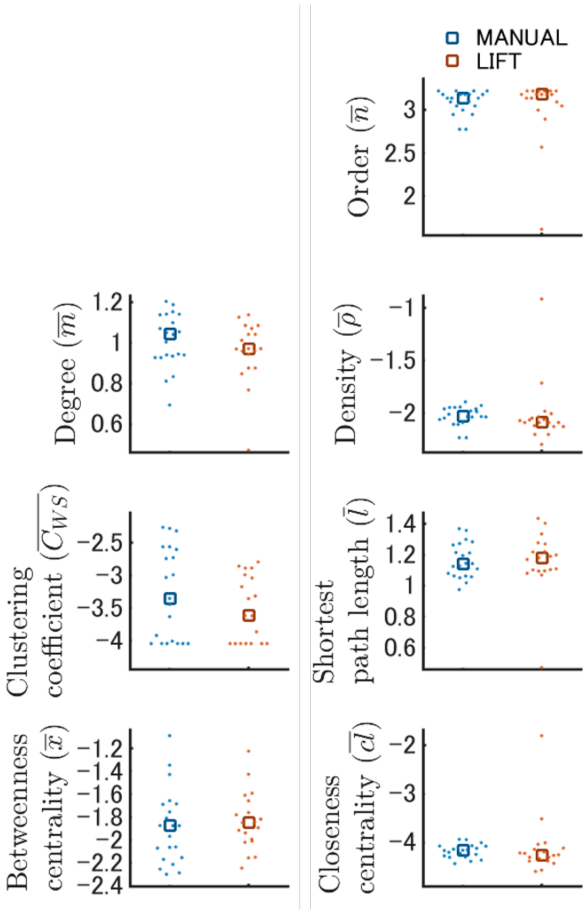


**Figure D. Network features generated by the static node generation method were comparable between the MANUAL and LIFT entries in the probe test.** The results of the MANUAL and LIFT entries in the probe tests on a log scale are shown in *blue* and *red*, respectively. Dots and squares indicate the raw and median values, respectively. Logarithmic transformation was performed for clearer viewing.

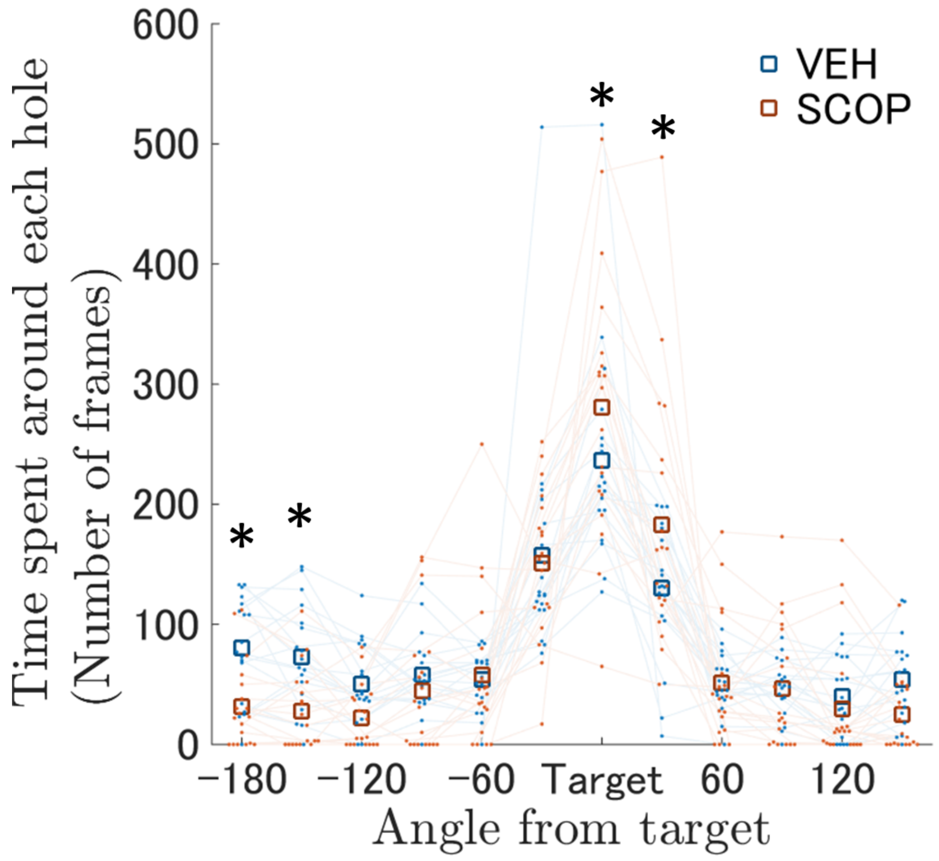


**Figure E. The probe test of VEH and SCOP groups for spatial reference memory in the Barnes maze.** The horizontal axis indicates the locations of the holes expressed as angle differences from the target. The vertical axis indicates the search time for the individual hole. The blue and red squares represent the mean results of the VEH and SCOP groups, respectively. The value of and change in each individual mouse's data are shown in dots and light lines. Mice in both groups spent significantly more time near the target hole compared with the other holes (See also Tables R–T in S1 File). The SCOP group mice spent slightly, but significantly, more time near the target area compared with the VEH group mice. Interestingly, the SCOP group mice spent significantly less time at −150 and −180 degree hole area than the VEH group mice (Table S in S1 File).

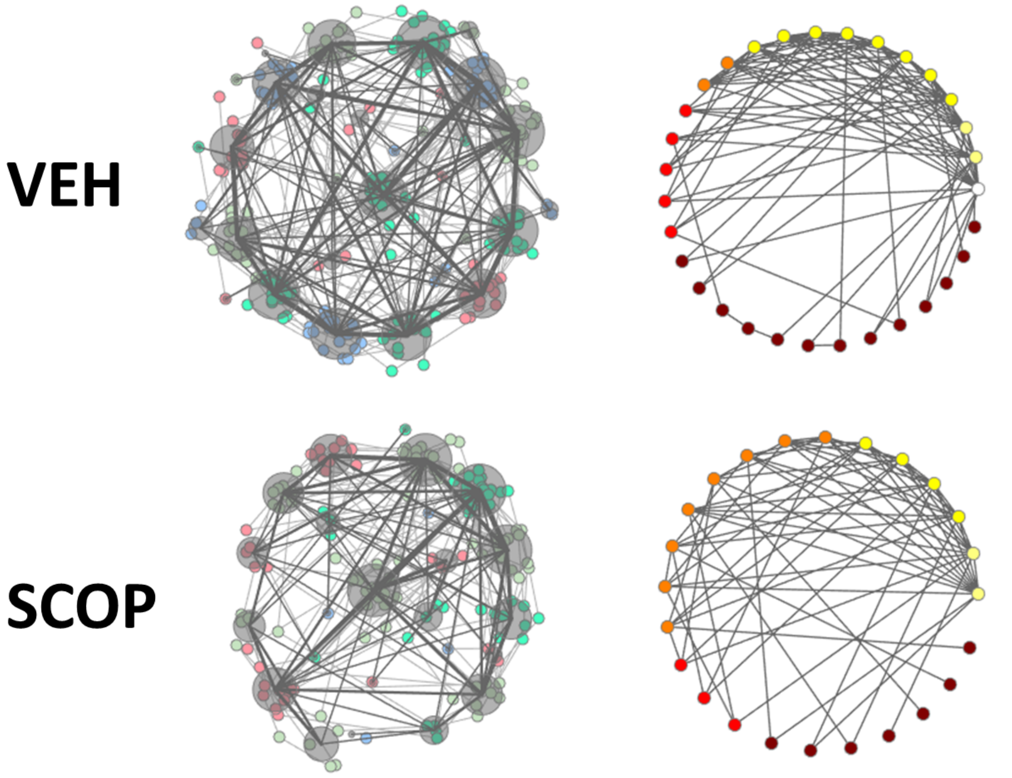


**Figure F. The exploratory network structures of the VEH and SCOP groups in the probe test.** Global network structures and their topological expression of search behaviors formed by the dynamic node generation method in the VEH and SCOP mouse groups are plotted in the upper and lower row, respectively. Although the both group mice generated remarlable nodes around the target, the SCOP mouse group tended to explore the more restricted area and generated nodes ununiformly. Compared with the more diversely interconnected link structure seen in VEH group, the small number of links was connected between the nodes located around the target area in SCOP group.

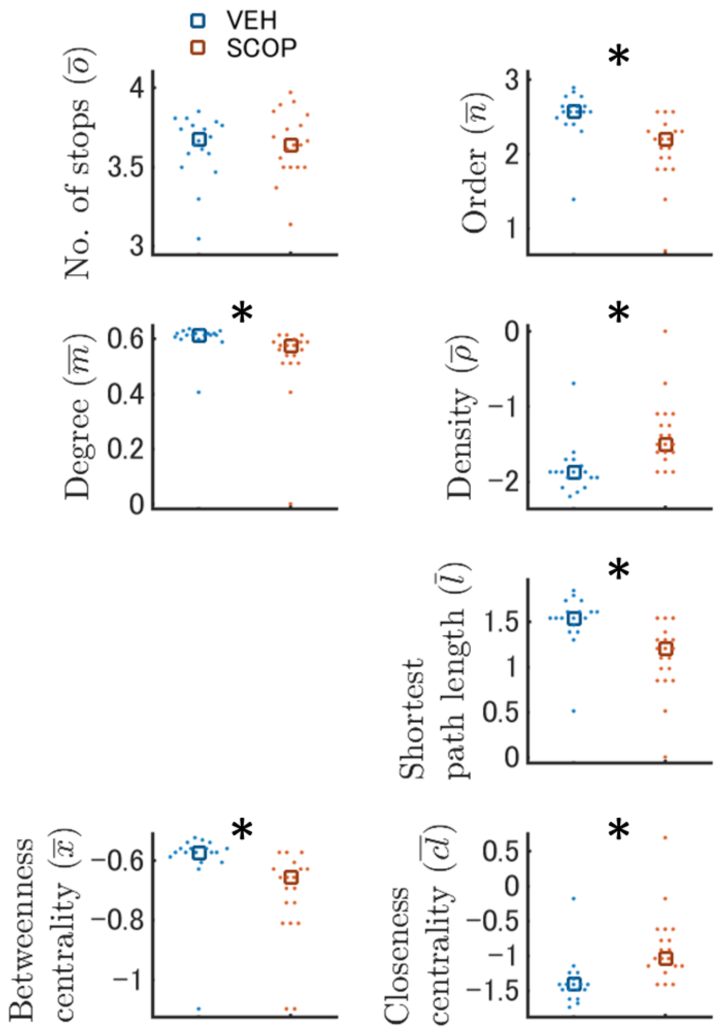


**Figure G. Structural difference in networks by dynamic node generation method between the VEH and SCOP groups in the probe test.** The results of the VEH and SCOP groups of the probe tests on a log scale are shown in *blue* and *red*, respectively. Dots and squares indicate the raw and median values, respectively.

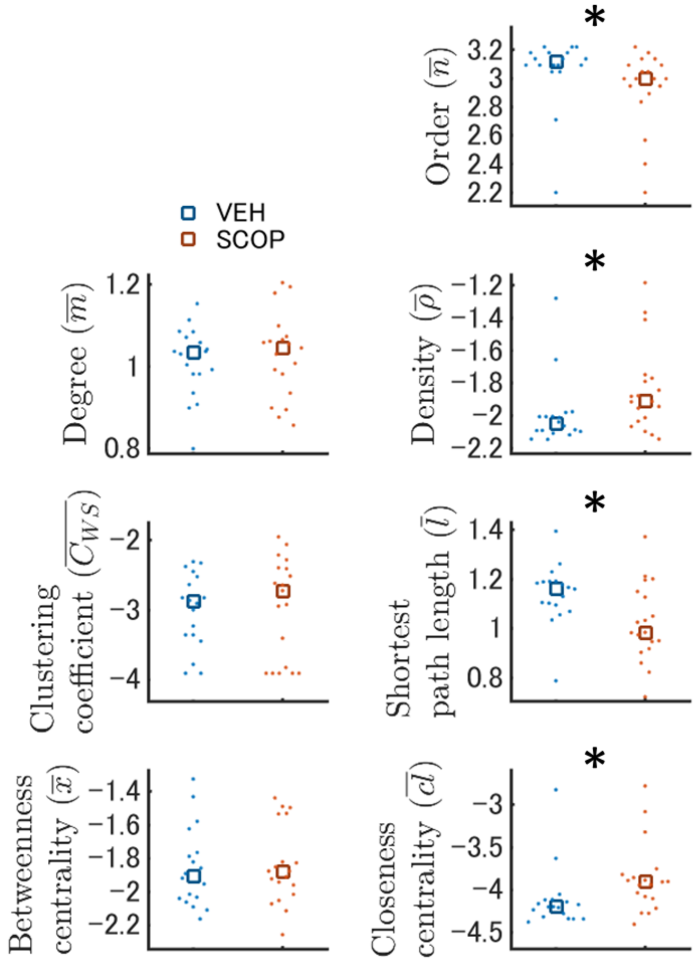


**Figure H. Structural difference in networks by static node generation method between the VEH and SCOP groups in the probe test.** The results of the VEH and SCOP groups of the probe tests on a log scale are shown in *blue* and *red*, respectively. Dots and squares indicate the raw and median values, respectively.
